# CD163 interacts with TWEAK to regulate tissue regeneration after ischaemic injury

**DOI:** 10.1038/ncomms8792

**Published:** 2015-08-05

**Authors:** Hirokuni Akahori, Vinit Karmali, Rohini Polavarapu, Alicia N. Lyle, Daiana Weiss, Eric Shin, Ahsan Husain, Nawazish Naqvi, Richard Van Dam, Anwer Habib, Cheol Ung Choi, Adrienne L. King, Kimberly Pachura, W. Robert Taylor, David J. Lefer, Aloke V. Finn

**Affiliations:** 1 Department of Internal Medicine, Division of Cardiology, Emory University, Atlanta, Georgia 30322, USA; 2 Division of Cardiology, Cardiovascular Center, Korea University Guro Hospital, Korea University College of Medicine, Seoul 152-703, Republic of Korea; 3 Kennesaw State University Department of Ecology, Evolution, and Organismal Biology Kennesaw, Georgia 30144, USA; 4 Atlanta VA Medical Center, Atlanta, Georgia 30033, USA; 5 Coulter Department of Biomedical Engineering at Georgia Tech and Emory, Atlanta, Georgia 30332, USA; 6 LSU Health Sciences Center, New Orleans, Louisiana 70112, USA

## Abstract

Macrophages are an essential component of the immune response to ischaemic injury and play an important role in promoting inflammation and its resolution, which is necessary for tissue repair. The type I transmembrane glycoprotein CD163 is exclusively expressed on macrophages, where it acts as a receptor for haemoglobin:haptoglobin complexes. An extracellular portion of CD163 circulates in the blood as a soluble protein, for which no physiological function has so far been described. Here we show that during ischaemia, soluble CD163 functions as a decoy receptor for TWEAK, a secreted pro-inflammatory cytokine of the tumour necrosis factor family, to regulate TWEAK-induced activation of canonical nuclear factor-κB (NF-κB) and Notch signalling necessary for myogenic progenitor cell proliferation. Mice with deletion of CD163 have transiently elevated levels of TWEAK, which stimulate muscle satellite cell proliferation and tissue regeneration in their ischaemic and non-ischaemic limbs. These results reveal a role for soluble CD163 in regulating muscle regeneration after ischaemic injury.

## Abstract (web summary)

CD163 is a glycoprotein receptor expressed on the surface of macrophages. Here, the authors demonstrate that a soluble form of CD163 can act as a decoy receptor for the pro inflammatory cytokine TWEAK, thereby revealing a new mechanism for the regulation of tissue repair after ischaemic injury.

## Graphical abstract

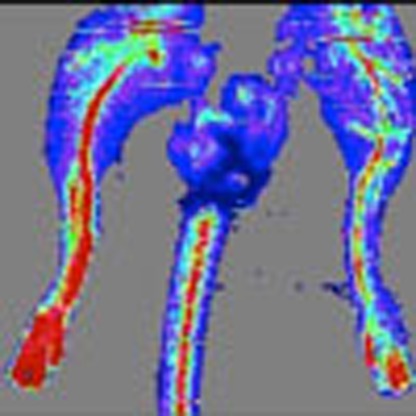

Vascular disease is the major cause of limb amputation^1^. Tissue hypoxia, resulting from narrowing of blood vessels, attracts macrophages that play a critical role in tissue repair^2^. Macrophages are a heterogeneous group of immune cells that respond to pathophysiological cues to form distinct functional phenotypes. In response to signals such as lipopolysaccharide and interferon gamma (IFN-γ), macrophages undergo classical M1 activation expressing high levels of pro-inflammatory cytokines, reactive oxygen species, and displaying high microbicidal and tumoricidal activity. M2 macrophages are stimulated by IL-4 and IL-13, express high levels of scavenging molecules, dampen pro-inflammatory cytokine levels and promote tissue remodelling^3^.

During tissue injury, M1 macrophages infiltrate the tissue first. Subsequent infiltration of M2 macrophages not only dampens M1 pro-inflammatory responses but also promotes tissue repair. Although M1/M2 dichotomy provides a conceptual framework for our understanding of the function of macrophages in the setting of injury, the mechanisms by which macrophages orchestrate inflammation and its resolution to promote tissue repair are incompletely understood.

M2 macrophages express high levels of CD163, a scavenger receptor expressed exclusively on cells of the monocyte/macrophage lineage^4^. CD163 functions as a receptor for haemoglobin:haptoglobin (Hb:Hp) complexes^5^. In addition, shedding of the extracellular domain of CD163 from macrophages increases plasma levels of soluble CD163 (sCD163)^6^. sCD163 shares ∼94% of the extracellular portion of membrane bound CD163, covering its nine scavenger receptor cysteine-rich domains^7^. Nevertheless, it displays relatively low affinity for Hb:Hp complexes, suggesting its function may be unrelated to haemoglobin scavenging^7^.

CD163 is generally considered an anti-inflammatory molecule involved with resolution of inflammation because pro-inflammatory stimuli such as lipopolysaccharide and oxidative stress suppress its expression and promote its shedding^7^,^8^,^9^. Moreover, CD163 expressing macrophages frequently are found in areas of regenerating tissues after a ischaemic injury^10^. However, the role of sCD163 in resolving inflammation and promoting tissue regeneration is uncertain.

One potential mechanism by which CD163 might orchestrate repair is through its interaction with the pro-inflammatory cytokine tumour necrosis factor-like weak inducer of apoptosis (TWEAK or TNFS12). TWEAK is mainly expressed by leukocytes, including macrophages^11^. It is both a membrane bound and soluble cytokine due to efficient cleavage by members of the furin protease family^12^. TWEAK acts by binding to Fn14, a highly inducible cell-surface receptor that is linked to several intracellular signalling pathways, including nuclear factor-κB (NF-κB), a pathway important in regulating responses to inflammation^13^. Fn14 is expressed at relatively low levels in many healthy tissues including tissue progenitor and endothelial cells^14^. It is upregulated, however, in the setting of injury, oxidative stress and inflammation^15^. In the setting of acute injury TWEAK has been suggested to play a beneficial role in promoting muscle repair through its effects on myogenic cells^16^. However, little is known about the biological mechanisms regulating TWEAK and the downstream pathways by which TWEAK might orchestrate muscle regeneration.

Here we show that during periods of tissue ischaemia sCD163 functions as a decoy receptor for TWEAK to regulate its ability to activate Notch signalling and stimulate myogenic progenitor cell proliferation. Loss of CD163 in mice subjected to unilateral femoral ligation resulted in transiently elevated levels of TWEAK, activation canonical NF-κB and Notch signalling and enhanced skeletal muscle regeneration not limited to the site of injury. These findings demonstrate a novel mechanism by which macrophages regulate tissue regeneration after ischaemic injury through control of the pro-inflammatory cytokine TWEAK.

## Results

### Systemic tissue regeneration after injury in CD163^−/−^ mice

**Figure 1:**
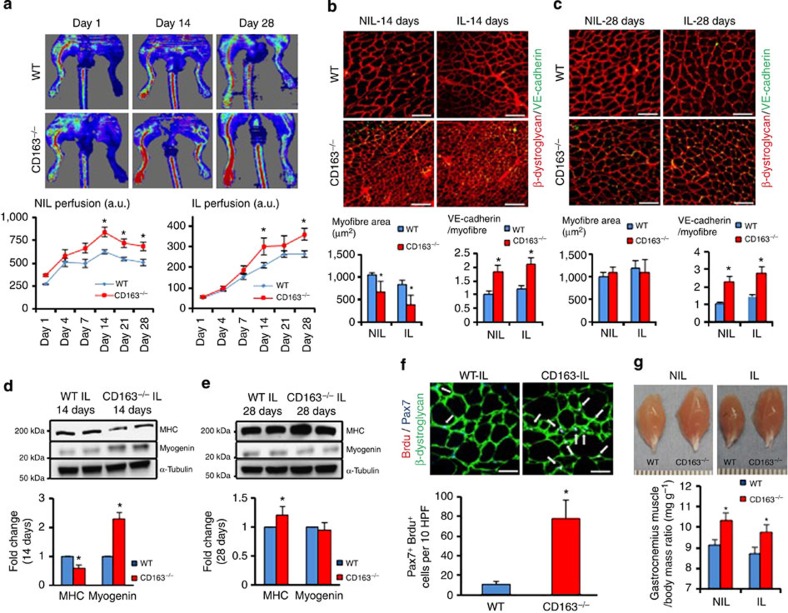
CD163^−/−^ mice exhibit enhanced skeletal muscle repair in both ischaemic and NILs in response to femoral ligation. (**a**) Laser Doppler analysis with perfusion quantitation 1, 4, 7, 14, 21 and 28 days after femoral ligation (*n*=10 per group). (**b,c**) Immunostaining of NIL and IL for VE-cadherin and β-dystroglycan (red) at 14 and 28 days after femoral ligation. The graphs below show the number of VE-cadherin positive cells/myofibre and the average muscle fibre area. (*n*=5 per each group). Scale bars, 100 μm. (**d**) Immunoblotting of IL at 14 days after femoral ligation (*n*=5 per group) with quantitation of densitometry for MHC and myogenin. (**e**) Immunoblotting of IL at 28 days after femoral ligation (*n*=5 per group) with quantitation of densitometry for MHC and myogenin. (**f**) Immunostaining of IL for β-dystroglycan, Pax7 (blue) and Brdu (red) at 14 days after femoral ligation with quantitation of average number of Pax7 Brdu positive cells (white arrows) per 10 high power field on right (*n*=5 per each group). (**g**) Weight of NIL and IL after femoral ligation (*n*=5 per group). Comparisons between two groups were achieved using a two-sided student's *t*-test. All bars show mean±s.e.m. *P<0.05 versus WT.

To define the role of CD163 in the host response to ischaemic injury, we subjected wild type (WT) and CD163^−/−^ mice to hind limb ischaemia (HLI). HLI was induced by unilateral femoral artery ligation. At baseline, WT and CD163^−/−^ mice not exposed to femoral ligation demonstrated no differences in limb LASER Doppler perfusion imaging (LDPI), capillaries (vascular endothelial cadherin (VE-cadherin) immunostaining) or muscle fibres (β-dystroglycan immunostaining) (Supplementary Fig. 1a,b). LDPI showed that 14–28 days after ligation, CD163 deficiency significantly increased blood flow in the ischaemic limb (IL) (Fig. 1a). Unexpectedly, CD163 deficiency also significantly increased blood flow to the contralateral non-ischaemic limb (NIL) in the same post-ischaemic period (Fig. 1a). Supporting this, immunostaining for VE-cadherin showed significant increases in the number of capillaries per muscle fibre in both limbs (Fig. 1b,c). Moreover, we found that the average size of muscle fibres in the gastrocnemius muscle, as determined by β-dystroglycan immunostaining, significantly decreased in both the IL and NIL of CD163^−/−^ mice at 14 days post ligation, whereas no changes were seen in WT mice (Fig. 1b,c). This difference in muscle morphology was no longer present by 28 days even though the number of capillaries remained higher (Fig. 1b,c).

To further characterize the response of CD163^−/−^ and WT mice to HLI, we examined levels of myogenin, a muscle-specific basic helix-loop-helix transcription factor important for myocyte (early skeletal muscle precursor) development and myosin heavy chain (MHC), an actin-based motor protein highly expressed in mature skeletal muscle. Immunoblotting of ischaemic gastrocnemius muscles at 14 days after HLI demonstrated a significant increase in myogenin and decrease in MHC in CD163^−/−^ mice, while at 28 days there was significantly more MHC but similar amounts of myogenin (Fig. 1d,e). Since these results suggest greater myogenic cell precursor (that is, myoblast) predominance in CD163^−/−^ mice at 14 days after HLI, we examined muscle stem cell proliferation using antibodies against bromodeoxyuridine (Brdu), a marker of cell proliferation and Pax-7, a marker for muscle satellite stem cells. Brdu+ Pax7+ cells were significantly and abundantly more present in 14 day IL of CD163^−/−^ versus WT mice (Fig. 1f). Consistent with these findings by 28 days after HLI both the IL and NIL of CD163^−/−^ mice weighed significantly more than that of WT mice (Fig. 1g). These results suggest stem cell-mediated muscle recovery is much greater in CD163^−/−^ mice by 28 days after HLI.

To complete our evaluation, we measured necrosis in the ischaemic gastrocnemius muscle and found it significantly reduced at 14 days in CD163^−/−^ mice (Supplementary Fig. 2a). Functional recovery was also examined on days 28 through 35 after HLI by measuring spontaneous running distance. There was a significant and impressive difference between CD163^−/−^ and WT mice in their running distance response to ischaemic insult while sham-operated CD163^−/−^ and WT mice ran a similar distance (Supplementary Fig. 2b). These findings suggest that in CD163^−/−^ mice femoral ligation results reduced tissue damage and enhanced functional recovery.

### Myeloid CD163 controls skeletal muscle regeneration after HLI

To determine whether myeloid CD163 deletion was responsible for the phenotype of CD163^−/−^ mice after femoral ligation we used bone marrow transplantation. We transplanted WT or CD163^−/−^ bone marrow cells into irradiated WT (referred to as WT→WT and CD163^−/−^→WT, respectively) or CD163^−/−^ (WT→CD163^−/−^ and CD163^−/−^→CD163^−/−^, respectively) recipients. We performed femoral ligation 6 weeks later. Compared with WT→WT and WT→CD163^−/−^, smaller but more numerous numbers of muscle cells associated with greater vascularity was observed in CD163^−/−^→WT and CD163^−/−^→CD163^−/−^ 28 days after HLI as indicated by staining for β-dystroglycan and VE-cadherin, respectively (Supplementary Fig. 3a,b). We further characterized skeletal muscle regeneration by performing immunoblotting of protein from the IL of transplanted mice for myogenin and MHC. As compared with WT→WT and WT→CD163^−/−^, greater myogenin but less MHC was observed in the skeletal muscle of CD163^−/−^→WT and CD163^−/−^→CD163^−/−^ mice (Supplementary Fig. 3c,d). These data demonstrate enhanced muscle progenitor cell responses after HLI is conferred by CD163 deficiency in bone marrow cells.

### Notch activation in CD163^−/−^ mice directs tissue regeneration

To understand the mechanism of enhanced regeneration in CD163^−/−^ mice, we examined retinal vascular patterning in postnatal day 6 (P6) WT and CD163^−/−^ mice. P6 CD163^−/−^ mice demonstrated increased vessel growth and stalk/connector cell formation, but decreased tip cell branching and filopodia (Supplementary Fig. 4a,b), a vascular pattern suggesting Notch activation^17^. To confirm this, we demonstrated significantly more lectin and Delta like ligand 4 (DLL4), a ligand for Notch receptors, double-positive cells in P6 retinas of CD163^−/−^ mice by immunohistochemistry (Supplementary Fig. 4c). Protein generated from whole CD163^−/−^ and WT P6 retinas also demonstrated increased levels of Notch intracellular domain (NICD), a proteolytic cleavage product formed after ligand-induced Notch activation, by immunoblotting (Supplementary Fig. 4d).

**Figure 2:**
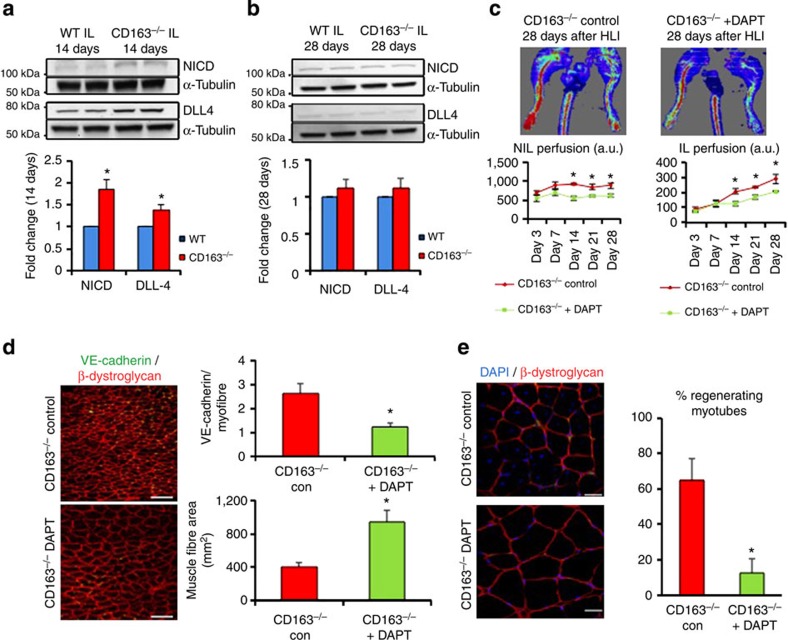
Notch activation in CD163^−/−^ mice after ischaemic injury directs muscle regeneration. (**a**) Immunoblotting of IL at 14 days after femoral ligation with quantitation of densitometry for NICD and DLL-4 (*n*=5 per group). (**b**) Immunoblotting of IL at 28 days after femoral ligation with quantitation of densitometry for NICD and DLL-4 (*n*=5 per group). (**c**) LASER doppler analysis of CD163^−/−^ mice 3, 7, 14, 21 and 28 days after femoral ligation with or without administration of Notch inhibitor DAPT (*n*=5 per group). (**d**) Immunostaining for VE-cadherin and β-dystroglycan (red) in CD163^−/−^ IL at 14 days after femoral ligation with or without DAPT administration. The graphs show the number of VE-cadherin positive cells/myofibre and the average muscle fibre area. Scale bars, 100 μm. (**e**) Immunostaining for β-dystroglycan (red) and TOTO-3 (blue) in CD163^−/−^ IL 14 days after femoral ligation with or without DAPT administration. Scale bars, 20 um. The graph shows quantitation of regenerating myotubes in CD163^−/−^ ILs with or without DAPT administration (*n*=5 per group). All bars show mean±s.e.m. *P<0.05 versus WT in **a**. versus CD163^−/−^ con in **c**,**d**,**e**. Comparisons between groups were achieved using a two-sided student's *t*-test.

Because Notch signalling appeared to play a major role in CD163^−/−^ mouse retinal vascular patterning and has been implicated in muscle progenitor cell proliferation, we examined Notch activation in WT and CD163^−/−^ mice 14 days after HLI^17^,^18^. Protein generated from the IL of CD163^−/−^ mice showed significant upregulation of DLL4 and increases in NICD by immunoblotting at 14 but not 28 days after HLI (Fig. 2a,b). Because these tissue homogenates consist mainly of skeletal muscle, we further confirmed Notch activation in endothelial cells 14 days after HLI by immunostaining the IL for DLL4 and VE-cadherin. There were significantly more DLL4/VE-cadherin double-positive cells in the IL of CD163^−/−^ mice (Supplementary Fig. 5). Both genotypes demonstrated no difference in Notch signalling at baseline as indicated by immunoblotting of skeletal muscle for NICD (Supplementary Fig. 1c).

Because Notch signalling has been implicated in myogenesis and is a context-dependent regulator of angiogenesis, we examined whether blocking Notch signalling using the γ-secretase inhibitor DAPT (*N*-[*N*-(3,5-difluorophenacetyl)-L-alanyl]-S-phenylglycine t-butyl ester) would inhibit the phenotypic responses of WT and CD163^−/−^ mice to HLI^17^,^18^. Treatment of CD163^−/−^ mice with DAPT completely inhibited the myogenic and angiogenic responses of CD163^−/−^ mice to HLI as indicated by LDPI, 14 day IL immunohistochemistry for VE-cadherin and β-dystroglycan, and the number of centrally located nuclei in myofibres (Fig. 2c–e). DAPT treatment had no effect on blood flow recovery in WT mice (Supplementary Fig. 6). Because Notch is not the only target of γ-secretase, we confirmed our observations using a blocking antibody against DLL4. The DLL4 blocking antibody also inhibited blood flow recovery and the angiogenic and muscle morphologic responses of CD163^−/−^ mice at 14 days after HLI (Supplementary Fig. 7). These data suggest that Notch signalling mediates the effects of CD163 deletion on myogenesis and angiogenesis after HLI.

Since Notch activation has been linked to changes in energy metabolism and tissue regeneration after ischaemic injury likely occurs in areas of hypoxia, we examined whether CD163 deficiency also led to changes muscle aerobic respiration^19^. We isolated mitochondria from IL and NIL of mice 14 days post HLI. A significant decrease in substrate oxidation was seen in State3 of CD163^−/−^ mitochondria isolated from IL and NIL 14 days after HLI compared with WT mice (Supplementary Fig. 8a,b,d,e), indicating suppression of mitochondrial respiration. Using quantitative PCR to analyse the IL after femoral ligation, transcripts consistent with enhanced glycolytic activity such as aldolase, galactose mutarose and hexokinase2 were significantly elevated in the IL and NIL of CD163^−/−^ mice (Supplementary Fig. 8c,f). There were no differences in either mitochondrial respiration or glycolytic transcripts of unligated mice (Supplementary Fig. 8g–i).

### CD163 deleted macrophages activate Notch signalling

Because CD163 is a monocyte/macrophage-specific protein, we investigated the phenotype and Notch activating ability of CD163^−/−^ bone marrow-derived macrophages (BMM). Compared with WT mice, BMM from CD163^−/−^ mice demonstrated increased transcription of Notch ligands and transcriptional targets including Notch-dependent M1 inflammatory genes such as *iNOS* and *IL-12* while markers of M2 macrophages such as mannose receptor c type 1 (mrc1) and PPARγ were unchanged (Supplementary Fig. 9a)^20^. Immunoblotting of protein from BMM demonstrated increased activation of NF-κB (that is, more degradation of Iκ-βα) and Notch (that is, NICD) (Supplementary Fig. 9b).

To determine whether BMM from CD163^−/−^ mice could activate Notch signalling in a paracrine fashion, cultured mouse dermal endothelial cells (MDEC) were exposed to supernatants from WT or CD163^−/−^ BMM for 3 h. As compared with WT supernatants, CD163^−/−^ supernatants stimulated Notch signalling as indicated by immunoblotting for NICD (Supplementary Fig. 9c).

### Canonical NF-κB signalling activates Notch in CD163^−/−^ mice

**Figure 3:**
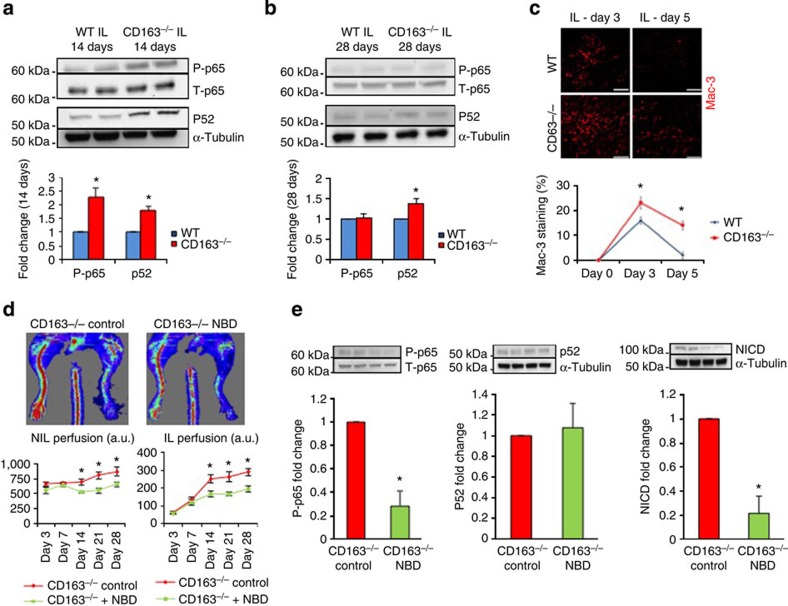
Canonical NF-κB signalling mediates Notch activation and the response of CD163^−/−^ mice to HLI. (**a**) Immunoblotting of IL 14 days after femoral ligation (*n*=5 per group) with graphs showing quantitation of densitometry for phosphorylated (p-p65)/total p65 (T-p65) and p52. (**b**) Immunoblotting of IL 28 days after femoral ligation (*n*=5 per group) with graphs showing quantitation of densitometry for phosphorylated (p-p65)/total p65 (T-p65) and p52. (**c**) Immunostaining for Mac-3 (red) of IL of WT and CD163^−/−^ mice 3 and 5 days after femoral ligation. Scale bars, 100 μm. The graph shows the timecourse of Mac-3 staining (*n*=5 per group). **(d)** Laser Doppler analysis of CD163^−/−^ mice 3, 7, 14, 21 and 28 days after femoral ligation with administration of scrambled control peptide (control) or NF-κB inhibitor NBD (*n*=5 per group). (**e**) Immunoblotting of CD163^−/−^ IL at 14 days after femoral ligation with administration of scrambled control peptide (control) or NF-κB inhibitor (NBD) (*n*=5 per group) with bar graphs showing quantitation of densitometry for phosphorylated/total p65, p52 and NICD on right. All bars show mean±s.e.m. *P<0.05 versus WT in **a**–**c**; versus CD163^−/−^ control in **d**,**e**. Comparisons between groups were achieved using a two-sided student's *t*-test.

Because NF-κB has been linked to Notch activation through upregulation of Notch ligands such as DLL4, we examined whether CD163^−/−^ mice exhibit increased NF-κB activity by examining both its canonical (p65/RelA) and non-canonical (p100/52) pathways^21^,^22^,^23^. Immunoblotting of IL and NIL 14 days after femoral ligation demonstrated increased p65 phosphorylation and p52 presence in CD163^−/−^ mice consistent with increased canonical and non-canonical activity while at 28 days only non-canonical signalling was activated (Fig. 3a,b). We then examined macrophage infiltration in the IL. Immunohistochemistry (that is, anti-Mac-3 staining) demonstrated greater macrophage accumulation and persistence in the CD163^−/−^ IL (Fig. 3c).

To confirm that Notch activation in CD163^−/−^ mice was mediated through NF-κB activation, we used the selective canonical NF-κB inhibitor NBD, which blocks the interaction of NEMO with IκB kinase complex^24^. NBD suppressed canonical but not non-canonical NF-κB signalling in CD163^−/−^ IL and inhibited the increased perfusion and Notch activation caused by CD163 deficiency as detected by LDPI and immunoblotting, respectively (Fig. 3d,e). NBD had no effect on blood flow recovery in WT mice (Supplementary Fig. 10). To rule out an independent effect of NBD on blood flow recovery or muscle morphology, CD163^−/−^ mice were subjected to sham procedure and treated with NBD or control for 14 days. No changes in either were seen (Supplementary Fig. 11). These results suggest canonical NF-kB signalling mediates Notch activation and the response of CD163^−/−^ mice to HLI.

### TWEAK mediates the response of CD163^−/−^ mice to HLI

**Figure 4:**
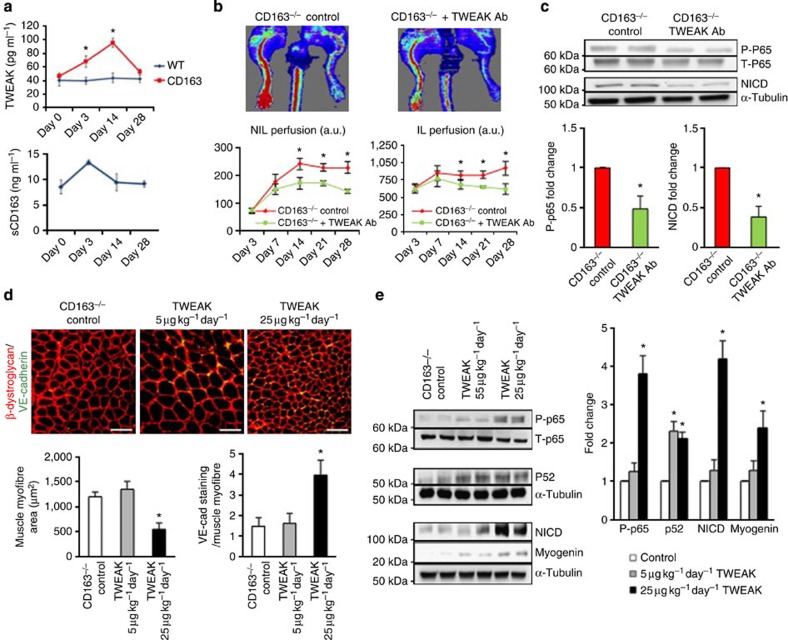
TWEAK activates canonical NF-κB signalling and Notch activation and mediates the response of CD163^−/−^ mice to HLI. (**a**) Analysis of plasma from WT and CD163^−/−^ mice before and after femoral ligation for TWEAK and sCD163 levels by ELISA (*n*=5 per group). (**b**) Laser doppler analysis of CD163^−/−^ mice 3, 7, 14, 21 and 28 days after femoral ligation administered an isotype control or TWEAK blocking antibody (*n*=5 per group). (**c**) Immunoblotting of CD163^−/−^ IL at 14 days after femoral ligation with administration of isotype control or TWEAK blocking antibody (*n*=5 per group) with graphs showing quantitation of densitometry for phosphorylated/total p65 and NICD below. (**d**) Immunostaining for VE-cadherin (green) and β-dystroglycan (red) in CD163^−/−^ limbs with 14 day administration of TWEAK (5 or 25 μg kg^−1^ day^−1^) or saline (control) (*n*=5 per group). Scale bars, 100 μm. The graphs show the number of VE-cadherin positive cells/myofibre and the average muscle fibre area. (**e**) Immunoblotting of CD163^−/−^ limbs with 14 day administration of TWEAK (5 or 25 μg kg^−1^ day^−1^) or saline (control) (*n*=5 per group) with graphs showing quantitation of densitometry for phosphorylated p65/total p65, p52 and NICD. All bars show mean±s.e.m. *P<0.05 versus WT in **a**; versus CD163^−/−^ control in **b**,**c** and **e**. 
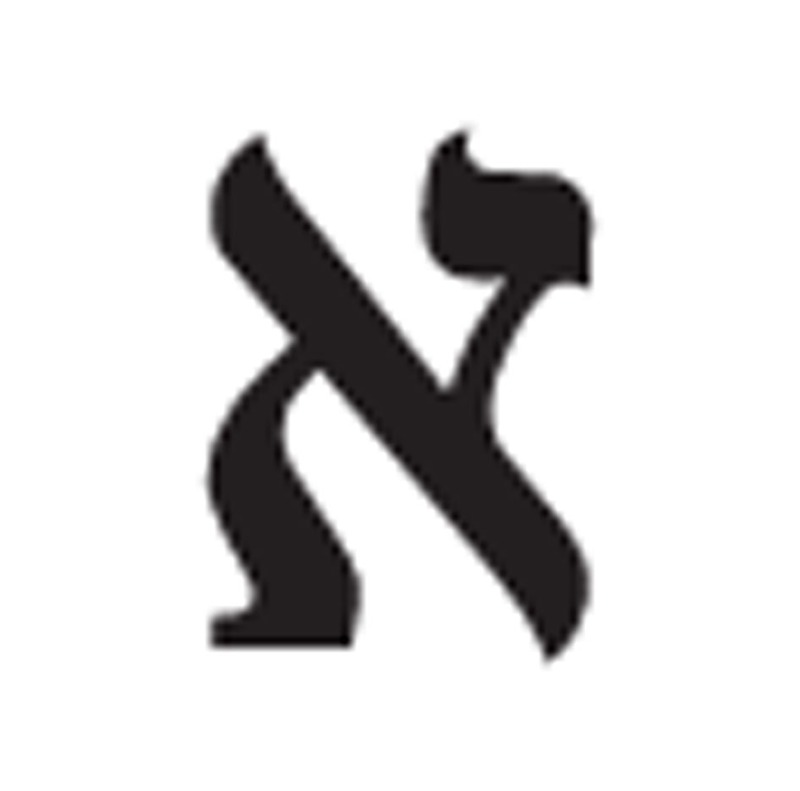
 *P*<0.05 versus other groups in **d**,**e**. Comparisons between groups were achieved using a two-sided student's *t*-test. For multiple group comparisons, we utilized a one-way ANOVA. If the variance ratio test (*F*-test) was significant, a more detailed *post hoc* analysis of differences between groups was made using a Tukey–Kramer honest significance difference test.

TWEAK is mainly expressed by leukocytes and becomes a soluble cytokine when cleaved by members of the furin protease family. TWEAK is known to induce NF-κB and in some contexts, such as cardiotoxin-induced muscle injury, promotes transient inflammation and may play a role in muscle regeneration^16^. Conversely, sustained TWEAK expression through transgenic overexpression can promote muscle pathology and impede the regenerative capacities of skeletal muscle^25^. Using a combinatorial peptide library, Bover *et al.*^26^ previously identified a potential protein–protein interaction between CD163 and TWEAK. We measured TWEAK and sCD163 concentrations in plasma obtained from both genotypes of mice after HLI. Compared with non-ligated WT and CD163^−/−^ mice and with WT mice after femoral ligation, the plasma of CD163^−/−^ mice after limb ligation contained significantly higher levels of TWEAK at 14 but not 28 days (Fig. 4a). Additionally, the amount of plasma sCD163 was significantly increased in WT mice 3 days after HLI (Fig. 4a). (Plasma sCD163 was not present in CD163^−/−^ mice.) Thus, CD163^−/−^ mice demonstrate transiently elevated levels of TWEAK, which contrasts with previous experiments in which chronic TWEAK overexpression has been linked to muscle degradation^25^.

To examine whether TWEAK was essential in the phenotype of CD163^−/−^ mice, we treated them with either a TWEAK blocking or an isotype control antibody after HLI. The TWEAK blocking antibody inhibited the increased perfusion caused by CD163 deficiency (Fig. 4b) but had no effect in WT mice (Supplementary Fig. 12). To confirm TWEAK was involved in the activation of canonical NF-κB signalling and Notch seen in CD163^−/−^ mice, immunoblotting for phosphorylated and total p65 and NICD of protein from the IL of control and TWEAK blocking antibody-treated mice was performed at 14 days post HLI. The TWEAK antibody prevented activation of both signalling pathways (Fig. 4c). These data provide evidence that TWEAK is responsible for Notch and canonical NF-κB activation in CD163^−/−^ mice after HLI.

### TWEAK infusion into CD163^−/−^ and WT mice

To explore whether TWEAK infusion could reproduce the effects seen in CD163^−/−^ mice at 14 days, we administered recombinant mouse TWEAK (Arg105-His249 with His Tag) or saline control into CD163^−/−^ naive mice and examined skeletal muscle 14 days later. TWEAK was given in two separate dosing strategies based upon previous work showing it to have a biphasic effect on cultured skeletal muscle with lower doses (10 ng ml^−1^) inducing non-canonical NF-κB activation and higher doses (100 ng ml^−1^) inducing both canonical and non-canonical pathways^27^. Low doses of TWEAK (5 μg kg^−1^ day^−1^) did not appreciably change skeletal muscle morphology or vascularity and activated only non-canonical NF-κB signalling (that is, p52) in skeletal muscle (Fig. 4d,e). By contrast, higher doses of TWEAK (25 μg kg^−1^ day^−1^) significantly increased skeletal muscle vascularity and decreased skeletal muscle size (Fig. 4d). Moreover, it also activated both canonical and non-canonical NF-κβ signalling, Notch activation, and increased myogenin as detected by immunoblotting of limbs (Fig. 4e). These changes are consistent with those seen in CD163^−/−^ muscle 14 days after HLI as well as previous data, in which muscle-specific transgenic overexpression of TWEAK-reduced muscle differentiation^25^. Our data suggest that high doses of TWEAK are able to stimulate muscle progenitor cell proliferation through activation of canonical NF-κB and Notch signalling, and thereby delay muscle differentiation. To determine whether CD163 is critical in mediating the biological effect of TWEAK, we administered low- and high-dose TWEAK to WT mice for 14 days. TWEAK had no effect on skeletal muscle morphology, vascularity or molecular signalling (Supplementary Fig. 13). Lastly, we determined whether high doses of TWEAK could also reproduce the changes in mitochondria seen in CD163^−/−^ mice after HLI. TWEAK infusion was able to decrease mitochondrial respiration in skeletal muscle. (Supplementary Fig. 14).

### TWEAK-mediated canonical NF-kB signalling activates Notch

**Figure 5:**
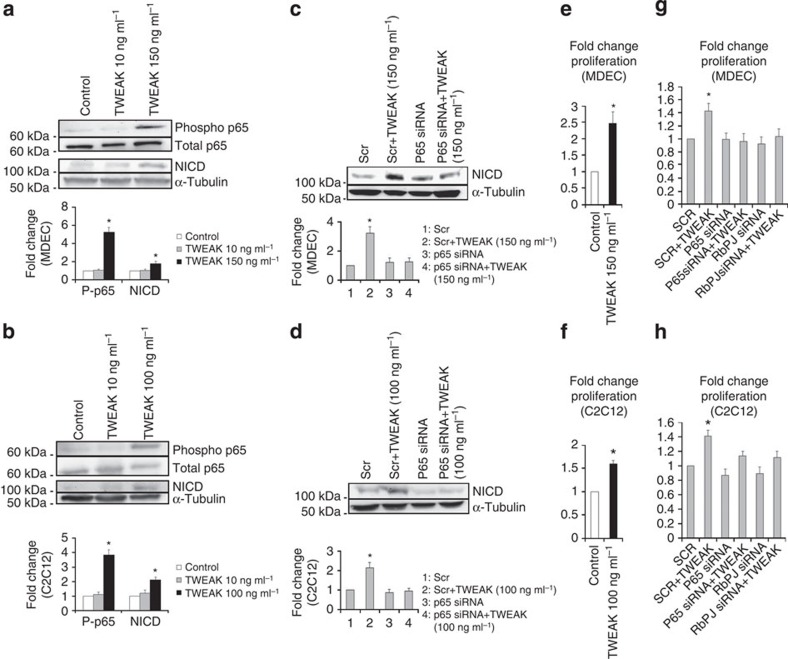
High-dose TWEAK activates canonical NF-κB and Notch signalling which stimulates cell proliferation in cultured endothelial and myoblast cells. (**a**) Immunoblotting of MDEC with or without administration of TWEAK (10 or 150 ng ml^−1^) (*n*=4 per group) for the indicated proteins. (**b**) Immunoblotting of C2C12 cells with or without administration of TWEAK (10 or 100 ng ml^−1^) (*n*=4 per group) for the indicated proteins. (**c**) Immunoblotting of MDEC transfected with scramble or p65 siRNA and treated with nothing or TWEAK (150 ng ml^−1^), (*n*=4 per group) for NICD. (**d**) Immunoblotting of C2C12 cells transfected with scramble or p65 siRNA and treated with nothing or TWEAK (100 ng ml^−1^) (*n*=4 per group) for NICD. (**e**) BrdU proliferation assay of MDEC treated with 150 ng ml^−1^ TWEAK (*n*=at least four per experiment repeated twice). (**f**) BrdU proliferation assay of C2C12 treated with 100 ng ml^−1^ TWEAK (*n*=at least four per experimental group repeated twice). (**g**) BrdU proliferation assay of MDEC treated with TWEAK (150 ng ml^−1^) in the presence of scramble siRNA or siRNAs against p65 or RBPJ (*n*=at least four per experimental group repeated twice). (**h**) BrdU proliferation assay of C2C12 treated with TWEAK (100 ng ml^−1^) in the presence of scramble siRNA or siRNAs against p65 or RBPJ (*n*=at least four per experimental group repeated twice). All bars show mean±s.e.m. *P<0.05 versus other groups. Comparisons between groups were achieved using a two-sided student's *t*-test. For multiple group comparisons, we utilized a one-way ANOVA. If the variance ratio test (*F*-test) was significant, a more detailed *post hoc* analysis of differences between groups was made using a Tukey–Kramer honest significance difference test.

To further confirm the effects of TWEAK on both MDEC and myoblast cells, respectively, we examined the effects of both low- and high-dose TWEAK on cultured MDEC and C2C12 cells. In both cells lines only higher doses of TWEAK-activated canonical NF-κB signalling and Notch activation (NICD) as detected by immunoblotting (Fig. 5a,b). Next, to confirm that canonical NF-κB signalling was responsible for Notch activation, we transfected cells with siRNA against p65, treated them with TWEAK and examined NICD by immunoblotting. Knockdown of p65 completely inhibited the activation of Notch signalling by TWEAK in both cell lines (Fig. 5c,d). Lastly, we evaluated the effect of TWEAK on MDEC and C2C12 proliferation and the effect of siRNA directed knockdown of p65 and RBPJ, a major transcriptional effector of Notch. TWEAK increased cell proliferation in both cell lines as detected by BrdU assay which was inhibited by treatment with siRNA against p65 and RBPJ (Fig. 5e–h). These results suggest high doses of TWEAK-induced stimulation of canonical NF-κB activates Notch signalling which mediates the proliferative muscle and endothelial responses seen in CD163^−/−^ mice at 14 days.

### sCD163 inhibits the effect of TWEAK

**Figure 6:**
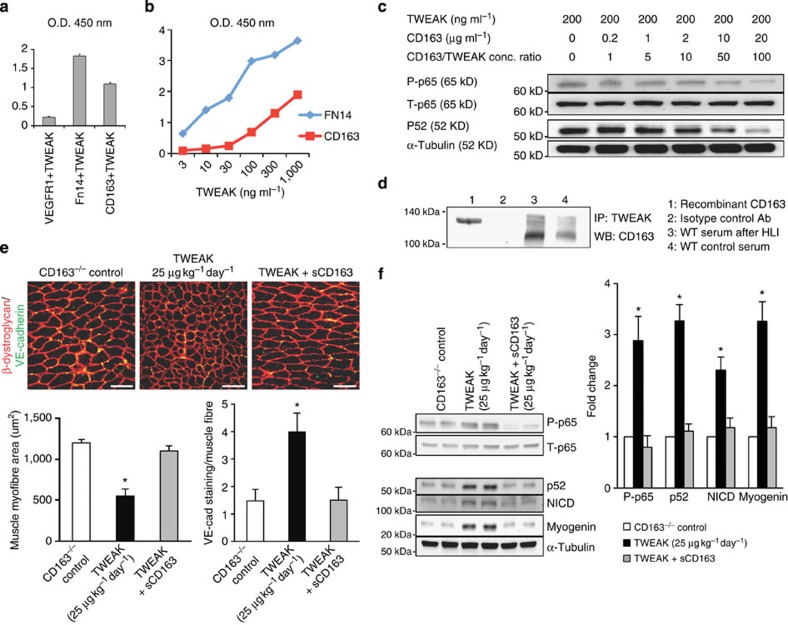
sCD163 inhibits the effect of TWEAK on tissue regeneration. (**a**) TWEAK (100 ng ml^−1^) was added to VEGFR1, Fn14 or CD163-coated wells. Bound protein was measured by ELISA using an antibody to detect TWEAK. (**b**) Fn14 or CD163 were coated onto 96-well plates and incubated with increasing doses of untagged TWEAK. Bound protein was measured by ELISA. (**c**) Immunoblotting of MDEC stimulated with TWEAK at 200 ng ml^−1^ (which activates both canonical and non-canonical NF-κB signalling) and increasing doses of recombinant mouse sCD163 for the indicated proteins. (**d**) Immunoprecipitation of serum before and after femoral ligation using anti-TWEAK antibody with immunoblotting for CD163. (The figures show a representative blot with a total of four experiments conducted.) (**e**) Immunostaining for VE-cadherin and β-dystroglycan (red) in limbs of CD163^−/−^ mice treated for 14 days with saline (control), TWEAK administration (25 μg kg^−1^ day^−1^ administered via Alzet pump) alone or in combination with sCD163 administration (25 μg kg^−1^ day^−1^ administered by intraperitoneal injection) (*n*=5 per group). The graphs show the number of VE-cadherin positive cells per myofibre and the average muscle fibre area per group. (**f**) Immunblotting of CD163^−/−^ limb after 14 days of TWEAK administration (25 μg kg^−1^ day^−1^ administered via Alzet pump) alone or in combination with sCD163 administration (25 μg kg^−1^ day^−1^ administered by intraperitoneal injection) (*n*=5 per group). O.D., optical density. Bar graphs show quantitation of densitometry for phosphorylated to total p65, p52 and NICD. *P<0.05 versus other groups. For multiple group comparisons, we utilized a one-way ANOVA. If the variance ratio test (*F*-test) was significant, a more detailed *post hoc* analysis of differences between groups was made using a Tukey–Kramer honest significance difference test. For binding experiments, average of at least four well per group shown with each experiment repeated at least two times with representative results shown.

Next we examined interaction between TWEAK and CD163 using a recombinant mouse CD163 (mCD163) protein which encompasses all nine scavenger receptor cysteine-rich domains of CD163 and thus mimics the structure of sCD163. We confirmed TWEAK binding abilities of CD163 via ELISA. Plates were coated with mCD163, mouse Fn14 (positive control) or mouse VE growth factor receptor 1 (VEGFR1, negative control). As seen in Fig. 6a, TWEAK demonstrated increased binding to both mCD163 and FN14 as compared with VEGFR1. Next, we evaluated whether TWEAK would bind to both CD163 and FN14 in a dose-dependent manner. Although TWEAK did bind to both receptors, it seemed to have slightly greater affinity for FN14 than CD163 (Fig. 6b). Next, we determined whether mCD163 was able to inhibit the biological effects of TWEAK on NF-κB signalling in mouse endothelial cells. Increasing doses of CD163 were added to endothelial cells in combination with TWEAK (200 ng ml^−1^). As seen in Fig. 6c, concentration ratios of CD163: TWEAK at or greater than 50 inhibited TWEAK-induced canonical and non-canonical NF-κB signalling. (Because previous data has shown that epitope tags on TWEAK might affect its biological behaviour (that is, ability to activate canonical NF-κB signalling pathways) via oligomerization, untagged recombinant mouse TWEAK was used for experiments in Fig. 6b,c (ref. 28). Overall no difference was found between His tagged and untagged recombinant mouse TWEAK in terms of their NF-κB activating ability in mouse endothelial cells). To investigate the interaction between CD163 and TWEAK *in vivo*, we immunoprecipitated TWEAK from the serum of mice before and after HLI and immunoblotted for CD163. Immunoprecipitation showed CD163/TWEAK complexes circulate in the serum of WT mice (Fig. 6d). Our recombinant sCD163 protein ran at a slightly higher molecular mass (∼130 kDa) than the native protein (110 kDA). This is consistent with heavy glycosylation as it is expressed in a mouse myeloma cell line (NS0 cells). To support a biological role for a TWEAK-CD163 interaction, we examined the effect of high-dose TWEAK infusion in CD163^−/−^ mice on skeletal muscle morphology and molecular signalling in the absence and presence of mCD163. mCD163 inhibited the increased muscle regeneration, NF-κB and Notch activation caused by TWEAK infusion alone (Fig. 6e,f). To rule out an independent effect of sCD163 on these end points we administered mCD163 to naïve CD163^−/−^ animals for 14 days and analysed muscle morphology and angiogenesis by immunohistochemistry for β-dystroglycan for VE-cadherin. sCD163 had no effect on either (Supplementary Fig. 15). These data indicate sCD163 is able to inhibit the biological effects of TWEAK.

## Discussion

Here we show that during periods of tissue ischaemia, macrophages release TWEAK to induce myogenic progenitor cell proliferation through activation of canonical NF-κB and Notch signalling. Subsequently sCD163 scavenges TWEAK to inhibit its ability to activate this pathway thereby promoting muscle differentiation. Loss of CD163 in mice resulted in transiently elevated levels of TWEAK-mediated activation of canonical NF-κB and Notch signalling in both the ischaemic and NILs which stimulated muscle satellite cell proliferation. Subsequent decreases in TWEAK and Notch signalling activation allowed progenitor differentiation which led to enhanced skeletal muscle regeneration not limited to the site of injury. Our findings highlight a novel mechanism by which macrophages coordinate tissue repair in ischaemia-injured skeletal muscle.

CD163 was first identified as a macrophage-specific scavenger receptor for Hb:Hp complexes^5^. In addition to its membrane bound form, a soluble variant of CD163 (that is, sCD163) exists in the plasma as well as other tissue fluids. sCD163 demonstrates relatively low affinity for Hb:Hp as compared with the membrane bound form, suggesting alternative functions for this receptor^7^. Using a combinatorial peptide library, Bover *et al.*^26^ first identified a potential interaction between CD163 and TWEAK. Although existing studies suggest that sCD163 may be a valuable diagnostic marker to monitor disease progression, definitive biological evidence for this interaction has been lacking in part due to lack of relevant animal model^29^. Here we use CD163-deficient mice to demonstrate for the first time an important biological role for sCD163 in the setting of tissue ischaemia. By binding to and inactivating TWEAK, sCD163 plays an essential role in (1) preventing TWEAK from mediating tissue regeneration in areas remote from the site of injury and (2) limiting the duration of TWEAK-induced activation of canonical NF-κB/Notch thereby limiting progenitor cell proliferation and promoting their differentiation. Recently, Fick *et al.*^30^ challenged the initial findings of Bover by demonstrating equal binding of recombinant TWEAK to CD163 expressing or empty vector transfected Hek293 cells. One potential and important difference between our own studies and those of Fick pertain the use of recombinant sCD163 peptide. Fick's assays mostly examined the ability of membrane bound CD163 to bind TWEAK rather than the soluble form. As demonstrated by Moller *et al.*^7^, the affinities of membrane bound and sCD163 for target proteins such as Hb:Hp are very different probably due to lack of receptor cross-linking (on the soluble version). Further studies of the affinity of membrane bound versus sCD163 for TWEAK are likely need to further investigate this.

TWEAK is both a membrane bound and a soluble pro-inflammatory cytokine known to activate NF-κB signalling through binding to its receptor Fn14 which is highly expressed on mesenchymal progenitor cells in including those of muscle lineage. Some studies have suggested that the membrane bound version of TWEAK as well as aggregates of soluble TWEAK have greater ability to activate canonical NF-κB signalling pathways as opposed to soluble TWEAK trimers^28^,^31^. Although we cannot rule out the involvement of membrane bound TWEAK in the changes seen after HLI in CD163-deficient mice, this seems less likely as exposure of mouse endothelial cells to cell free supernatants from CD163-deficient mouse macrophages were able to activate Notch signalling (which requires canonical NF-κB activation) (Supplementary Fig. 9c). However, given the relatively low plasma concentration of serum soluble TWEAK (100 pg ml^−1^) after HLI and the low affinity of sCD163 for soluble TWEAK, it remains possible that macrophage expressed CD163 acts by inhibiting macrophage expressed membrane TWEAK. To what extent aggregates of TWEAK might play a role in its biological activity *in vivo* remains unclear and deserves further investigation.

**Figure 7:**
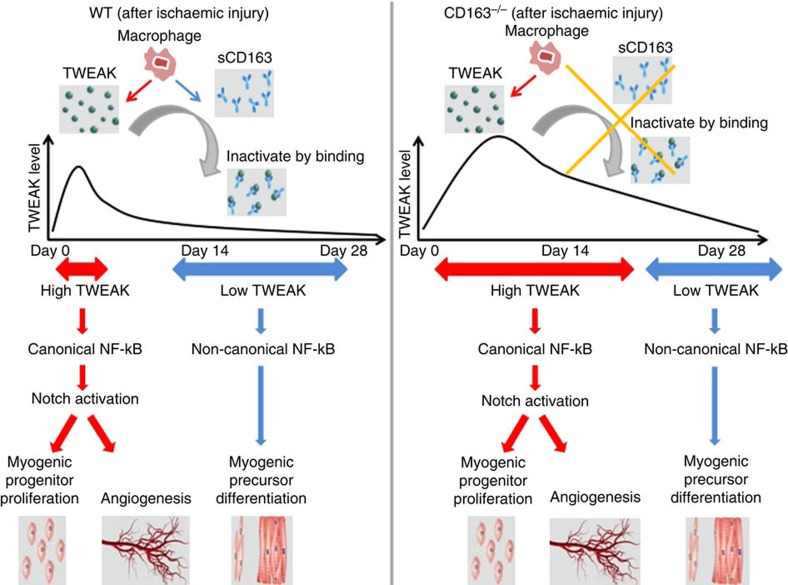
CD163-TWEAK interaction controls skeletal muscle recovery after ischaemic injury. After ischaemic injury, macrophages secrete TWEAK which induces tissue canonical NF-KB/Notch mediated myogenic progenitor cell proliferation. TWEAK's biological activity is tightly regulated by sCD163 binding which limits serum TWEAK levels and leads to resolution of canonical NF-κB/Notch activation at the site of injury, allowing for cell differentiation. In the absence of sCD163 (as seen in CD163-deficient mice), TWEAK levels are systemically elevated and TWEAK's biological effect is unopposed leading to prolonged activation of NF-κB and Notch signalling which causes greater myogenic progenitor cell proliferation-induced myogenesis not limited to the site of injury.

Previous studies of TWEAK in the context of muscle injury have shown varying effects with some suggesting TWEAK may contribute to muscle repair in the setting of an acute injury^16^. Other studies demonstrate opposite effects of TWEAK in the setting of chronic TWEAK overexpression which appeared to inhibit the regenerative capacities of muscle tissue^25^. More recent studies even suggest TWEAK impedes muscle renewal through inhibition of Notch signalling and satellite cell numbers in myofibre cell explants^32^. How do we resolve these apparent discrepancies with our own findings which suggest enhanced muscle repair in the setting of ischaemic injury in CD163-deficient mice? In the present study, we demonstrate the importance of sCD163 in determining TWEAK levels and biological activity. Transient upregulation of TWEAK induced by CD163 deficiency at day 14 after HLI increased canonical NF-κB and Notch activation which was associated with myogenic progenitor cell proliferation. By 28 days TWEAK levels, Notch activation, and canonical NF-κB signalling were no longer different between WT and CD163-deficient mice although non-canonical signalling remained higher (Fig. 7). This appears to have allowed the differentiation of early myogenic progenitors (that is, myoblasts) into mature cells as indicated by histology, MHC levels, and prior data which supports a role for non-canonical NF-κB signalling in myoblast fusion^27^. Although we did not specifically examine the effect of lower doses of TWEAK on satellite cell behaviour, Ogura *et al.*^32^ recently found that lower doses of TWEAK (10 ng ml^−1^) actually inhibited some Notch transcriptional targets such as Hey1 and HeyL in cultured myoblasts. We did not detect any changes in Notch activation as measured by NICD in both the *in vitro* and *in vivo* settings under similar dosing conditions, although it remains possible that lower doses of TWEAK might repress Notch signalling to promote myogenic precursor cell differentiation. This is not inconsistent with our findings in CD163^−/−^ mice that decreasing levels of TWEAK between 14 and 28 days after HLI coincided with muscle differentiation as evidence by increases in MHC. We also do not dispute that more prolonged TWEAK expression, as shown by others, might impede regenerative capacities of muscle through mechanisms that likely involve prolonged NF-κB-mediated inflammation and mitochondrial dysfunction.

In addition, recent findings in TWEAK knockout mice in the setting of muscle injury deserve to be addressed. Mittal *et al.*^25^ found that genetic deletion of TWEAK increased muscle fibre cross-sectional area and levels of MHC in regenerating muscle, while myoblasts from mice with muscle-specific transgenic overexpression of TWEAK showed reduced differentiation. This data is not inconsistent with our findings that demonstrate high doses of TWEAK promote muscle proliferation and delay differentiation (as seen in CD163^−/−^ mice 14 days after HLI or after high-dose TWEAK infusion), while its absence likely promotes muscle differentiation (similar to what we see 28 days after HLI in CD163^−/−^ mice). Ogura *et al.*^32^ also reported that TWEAK knockout mice demonstrate increased Pax7^+^ cells and increase in some Notch transcriptional targets after cardiotoxin-induced injury, findings which would seem to contradict our own work suggesting high doses of TWEAK activate Notch signalling and Pax7 cell proliferation. However, one important caveat of Ogura *et al.*^11^ findings is the reduced overall inflammation seen in TWEAK-deficient mice leaves open the possibility that inflammatory mediators other than TWEAK might be responsible for these findings.

A link between NF-κB and Notch signalling has been suggested by others in the setting of cancer as well as in mice with mutant endothelial cell-specific NF-κB (dominant negative Iκ-βα)^22^,^23^. How does canonical NF-κB signalling activate Notch pathways? Although not specifically explored here, the effect of NF-κB on Notch activation may in part be mediated through increased expression of the Notch ligand DLL4 and Notch1 receptor each of which has a canonical NF-κB binding motif (5-GGRRNNYYCC) in their promoters. Further support for this concept is suggested by our data showing that a specific DLL4 blocking antibody could inhibit the angiogenic and regenerative responses of CD163 mice to HLI.

The effect of Notch signalling in postnatal myogenesis has been well-studied and demonstrates satellite cell activation, proliferation and cell lineage determination are regulated by Notch signalling^18^. Thus our results that activation of Notch in muscle tissue is associated with satellite cell proliferation and myoblast predominance at 14 days after HLI is in accordance with these results. The role of Notch signalling in postnatal vascular homeostasis is less well understood. In retinal vascular development endothelial cells within a developing blood vessel sprout utilize the Notch signalling pathway to coordinate cellular behaviours during angiogenesis^17^. Notch receptor activation appears to occur primarily in ‘connector' or stalk cells while ‘tip' cells locating at the leading edge of an angiogenic front migrate and branch. In various models of angiogenesis including HLI, inhibition of Notch signalling promotes vascular sprouting, failure of tip cell fusion and dysfunctional vasculature^23^,^33^. Although we did not specifically measure vascular branching in the HLI model, our data suggest that Notch activation in endothelial cells can promote the development of functional vasculature. CD163-deficient animals receiving the Notch inhibitor DAPT or an anti-DLL4 blocking antibody demonstrate inhibition of blood flow recovery and angiogenesis after HLI and underscore that Notch signalling in endothelium of adult mice can promote functional angiogenesis.

Our findings reveal the importance of macrophage sCD163 in regulating tissue regeneration after ischaemic muscle injury. Loss of CD163 in mice resulted in transiently elevated levels of TWEAK-mediated activation of NF-κB and Notch signalling in both the ischaemic and NILs of CD163^−/−^ mice which led to enhanced myogenesis/angiogenesis not limited to the site of injury. The increased NF-κB and Notch activation seen in the ischaemic and NILs of these mice did resolve by 28 days after HLI, leading to cellular differentiation and enhanced regeneration. These data highlight the importance of both inflammation and its resolution in proper tissue regeneration and critical role of the CD163-TWEAK interaction in this process. The CD163-TWEAK pathway we have identified in the setting of limb ischaemia may be a novel target for regenerative therapies.

## Methods

### Animals

The Institutional Animal Care and Use Committee at Emory University approved all animal protocols. All animal experiments were conducted according to the National Institutes of Health Guide for the Care and Use of Laboratory Animals. CD163 knockout mice (CD163^tm1(KOMP)Vlcg^) were generated using targeting constructs available from the University of California at Davis International Mouse Consortium (KOMP). Mice were generated by the Emory transgenic mouse core on a C57BL6 background. WT (that is, C57BL6) mice used for all experiments were littermate controls. Male mice 8 weeks of age were used for all experiments.

### Experimental animal protocols

For studies of Notch signalling, WT or CD163^−/−^ mice were randomized in two groups: mice subjected to intraperitoneal administration of carrier (controls, *n*=5); or Notch inhibitor DAPT (10 mg kg^−1^ day^−1^, *n*=5) every other day for 14 or 28 days after HLI. DAPT was initially dissolved in dimethylsulphoxide to make a stock solution and then resuspended in 10% ethanol 90% corn oil as previously described^34^. For experiments involving inhibition of Notch signaling with a DLL4 blocking antibody (BioLegend) or the LEAF-purified DLL4 blocking antibody (BioLegend), antibodies were administered at an approximate dose of 250 mg twice weekly by intraperitoneal injection for 14 or 28 days after HLI. The dosing was based upon previous studies^35^. For experiments involving inhibition of NF-kB signalling, CD163^−/−^ mice were randomized into two groups: mice subjected to intraperitoneal administration with IKK-NBD control peptide (100 μg day^−1^, *n*=5, Enzo Life Sciences); or NF-kB inhibitor NBD (100 μg kg^−1^ day^−1^, *n*=5, Enzo Life Sciences) daily for 14 days after HLI. For LDPI experiments using NBD, animals received either control peptide or NBD (100 μg kg^−1^ day^−1^) via Alzet pump (Model 2800) for 28 days. Dosing was based upon previous studies^24^. For experiments involving inhibition of TWEAK signalling, CD163^−/−^ mice were randomized into two groups: mice subjected to intraperitoneal administration with LEAF-purified isotype control (Biolegend, *n*=5), or TWEAK blocking antibody (BioLegend clone MTW-1, 1 mg kg^−1^ day^−1^, *n*=5) daily for 14 days after HLI. For LDPI experiments using isotype control or TWEAK blocking antibody, animals received either control or anti-TWEAK antibody (1 mg kg^−1^ day^−1^) intraperitoneally for 28 days. Dosing was based upon manufacturers recommendation. For studies involving recombinant mouse TWEAK administration, WT or CD163^−/−^ mice were randomized into three groups: mice subjected to administration of phosphate-buffered saline (PBS) (control) via Alzet pump (Model 2800), mice subjected to administration of low dose TWEAK via Alzet pump (5 μg kg^−1^ day^−1^, *n*=5), or high-dose TWEAK (25 μg kg^−1^ day^−1^, *n*=5) via Alzet pump. For studies involving administration of CD163 peptide with and without recombinant high-dose mouse TWEAK-CD163^−/−^ mice were administered either high-dose TWEAK as described above or both mCD163 (25 μg kg^−1^ day^−1^ delivered by once daily by intraperitoneal injection) and high-dose TWEAK via Alzet pump (Model 2800). This resulted in concentration ratios of CD163: TWEAK of ∼89 as determined by ELISA. For experiments involving administration of NBD into sham-operated CD163^−/−^ mice or mCD163 into naive mice dosing was as stated above. Animals were randomly assigned to study groups for *in vivo* studies; no formal randomization method was applied when assigning animals for treatment. No blinding was used.

### BM transplantation

Bone marrow transplantation was conducted as previously reported^36^. Briefly, bone marrow was obtained from 8-week-old CD163^−/−^ and littermate WT donor mice by flushing femurs and tibiae with RPMI 1640 medium. Recipient mice were 8-week-old CD163^−/−^ or WT mice. Recipients were irradiated with 1,100 rads and then were immediately reconstituted with either 2 × 10^6^ CD163^−/−^ or WT bone marrow cells via retro orbital injection. Eight weeks later, spleen samples of some recipient CD163 mice were analysed for western blotting.

### Hind limb ischaemia model

At 8 weeks of age, animals were anesthetized with 2% isofluorane through a nose cone. Using aseptic technique, left superficial femoral artery was ligated proximal to the caudally branching deep femoral artery and proximal to the branching of the tibial arteries. The length of the artery was excised between the two ligation points, leaving the femoral nerve intact. The skin was closed with monofilament sutures. To measure blood flow, LDPI was performed as described previously^37^. Briefly, mice were anesthetized by inhalation of 2% isofluorane and scanned with the LDPI system (PIM II Laser Doppler Perfusion Imager). Functional recovery of the IL was assessed by monitoring of spontaneous activity in cages equipped with a running wheel.

### Histology and Immunohistochemistry

We injected 10 ml of Dulbecco's PBS/Heparin (10 unit per ml), 10 ml of vasodilation solution (100 μM adenosine, 10 μM sodium nitroprusside and 0.05% bovine serum albumin directly dissolved in Dulbecco's PBS with Ca^2+^Mg^2+^) and 10 ml of 4% paraformaldehyde solution consecutively at a rate of 10 ml min^−1^ into left ventricle of the heart. Gastrocnemius muscles were collected after perfusion fixation. Tissue samples were transferred into 15% sucrose solution and incubated at 4 °C for 2–3 h, followed by incubation in 30% sucrose solution at 4 °C overnight. Tissue samples were embedded in optimal cutting temperature compound and frozen, followed by sectioning at 15 μm thickness for limb samples. Sections were incubated in primary antibodies β-dystroglycan at a dilution of 1:250 (cat. #: ab49515 Abcam), Mac-3 at a dilution of 1:100 (Cat #: 553322 BD Pharmingen), VE-cadherin 1:250 (cat #: ab33168 Abcam), DLL4 at a dilution of 1:50 (cat #: AF1389, R&D Systems), Pax7 at a dilution of 1:250 (Cat No: ARP32742, Aviva Systems Biology) for 24 h at 4 °C and fluorescently labelled secondary antibody if required donkey anti-mouse IgG 546 nm (Cat. No: A10036, Invitrogen) and donkey anti-mouse IgG 488 nm (Cat. No: A21202, Invitrogen). Tissue samples were mounted on slides with Vectashield mounting medium (Vector Labs, Burlingame, CA). TOTO-3 (Invitrogen) was used as a nuclear counterstain (Invitrogen). Tissue was visualized using objective on a Nikon Eclipse TE- 2000U microscope (Nikon, Tokyo, Japan) with HeNe laser, and driven by EZ-C1 Viewer v3.5 software (Nikon). Projection images were generated by collecting the maximum pixel intensity from each image of the Z stack and by projecting pixel intensity onto the single (projection) image. The area and percentage of staining was quantified using NIS-elements v3.0 (Nikon). Staining area was quantified using computer-aided planimetry and was expressed as a percentage of the total surface area of the tissue section. The area of gastrocnemius muscle was quantified using by manually outlining cells with contiguous β-dystroglycan. Necrotic area in the gastrocnemius muscle was analysed by haematoxylin and eosin staining. Necrotic cells display a more glassy homogeneous appearance in the cytoplasm with increased eosinophilia.

### Analysis of limb regeneration

BrdU (100 mg kg^−1^ cat #: 2760 Millipore) was injected intraperitoneally every day from 7 to 14 days after HLI. Gastrocnemius muscles were removed 14 days. Immunofluorescence was performed using antibodies against BrdU, β-dystroglycan and Pax7 as described above. Data was expressed as the average number of BrdU Pax7 positive cells per 10 high power fields. As a measure of regeneration efficacy, we also counted the number of centrally nucleated regenerating myofibres 14 days after HLI.

### Immunoblotting and quantitative PCR with reverse transcription

Protein was processed and separated on polyacrylamide gel as previously reported^38^. Blot membranes were incubated with commercially available antibodies against DLL-4 (cat# AF1389 R&D Systems), NICD (cat #4147 Cell Signalling), Ikβα (#4882 Cell Signaling), α-tubulin (Cell Signaling cat #2125), phospho-NF-κB p65 (cat #3039 Cell Signaling), Total p65 (cat #4764 Cell Signaling ), p100/52 (cat #4882 Cell Signaling), MHC-MF20 (Developmental Studies Hybridoma Bank, Iowa City, IA) and myogenin-F5D (Developmental Studies Hybridoma Bank, Iowa City, IA). For loading controls, the membranes were stripped and reprobed with α-tubulin (cat # 2125 Cell Signaling). Proteins were visualized using the SuperSignal chemiluminescence reagent substrate(Pierce) and Biorad Chemdoc system. Densitometry, that is, area × density analysis was quantified with Quantity One 4.5.2 1-D Analysis Software (Bio-Rad, Hercules, CA). For each experimental group, a ratio of phosphorylated protein to total protein or a ratio of each protein to α-tubulin was calculated and normalized to control.

Total RNA was isolated using Qiazol (Qiagen) and RNeasyMini Plus kit (Qiagen, Valencia, CA). The yield and purity of the total RNA was assessed by measuring the absorbance at OD260 and OD280 with the Nanodrop 2000 (Thermoscientific). The ratio of A260/A280 for all RNA samples was between 1.8 and 2.0, which indicated that the quality and purity of RNA was adequate for subsequent cDNA synthesis. The extracted total RNA was reverse transcribed to first strand cDNA using High Capacity RNA-to-cDNA kit (Life Technologies, Carlsbad, CA). PCR and analysis were performed on the StepOne Plus detection systems and software (Life Technologies). Each sample was run in triplicate. Data normalization was accomplished by using 18S or GAPDH as the endogenous control. The relative expression of target genes was expressed as fold change between reference control and experimental groups. The primer pairs used were, mouse 18S: sense CCCTGCCCTTTGTACACACC and antisense CGATCCGAGGGCCTCACTA and mouse FN14: sense GACCTCGACAAGTGCATGGA and antisense CGCATCCCAGGCAGAAGT. PCR was performed using Fast SYBRG. For mRNA analysis of Aldob (sense: AGA GCA TCG GCG GAG TGA, antisense: TTT CCC TGG CTG TCC TTC TG), Galm (sense: TGC TTG GCT TTG CGG AAT, antisense: CCC AAC CAC TGC TCC AAA GT) Hk2 (sense: GCC AAG CGT CTC CAT AAG G, antisense: CGG AGG AAG CGG ACA TCA) and M1 and M2 transcripts Taqman Inventoried gene expression probes were ordered from Life Technologies. PCR reactions were performed using TaqMan Fast Universal PCR Master Mix.

### Mitochondria isolation and respiration measurements

Mitochondria were isolated from the ischaemic and NIL of WT and CD163^−/−^ mice after HLI or from one limb after high-dose TWEAK infusion into CD163^−/−^ mice by differential centrifugation techniques^39^. Oxygen consumption of isolated mitochondria was monitored using a Clark-type oxygen electrode (Hansatech Instruments, Amesbury, MA). Respiratory capacity was assessed by measuring State 3 (that is, ADP-dependent) and State 4 (that is, ADP-independent) respiration, using glutamate/malate as the oxidizable substrate. The respiratory control ratio was calculated as the ratio of states 3 and 4 respiration rates (i.e., states 3 divided by 4 respiration).

### Bone marrow-derived macrophage (BMM) isolation

Eight-week-old CD163^−/−^ mice and littermate WT mice were used for bone marrow extraction and subsequent macrophage isolation. Muscle tissue was removed from the bones using scissors and bones were cut at both ends to free the marrow. Bones were flushed with RPMI (+glutamine) medium supplemented with antibiotics. The bone marrow cells were triturated to bring the cells into single cell suspension. The slurry was then filtered using a sterile 70 μM nylon strainer to remove debris. Cells were centrifuged at 200*g* for 3 min. The supernatant was aspirated, and the cells were resuspended in RBC Lysis buffer (eBioscience) to lyse the RBC for 5 min. The cells were then resuspended and washed three times with BMM medium (that is, RPMI medium with glutamine (cat#11875-093 Life Technologies), supplemented with 10% FBS (Atlanta Biologicals) and 1% Penicillin/Streptomycin (Life Technologies)). Bone marrow cells were counted using an automated cell counter. The cell concentration was adjusted to 4 × 10^7^ cells ml^−1^ in BMM medium. Cells were seeded in a six-well plate (Falcon) and incubated in a humidified incubator with 5% CO_2_ at 37 °C. Media was changed twice, at 6 and 24 h after seeding, at which time media was supplemented with 40 ng ml^−1^ macrophage colony-stimulating factor (m-CSF) (cat#14-8983 eBioscience). Cells were incubated at 37 °C and differentiated into macrophages in BMM-mCSF medium for 7 days.

*Cell culture*. Mouse Dermal microvascular Endothelial Cells (MDEC) (cat#C57-6064 Cell Biologics) were used at passages 3 through 8. Cells were grown in complete growth medium (CGM) (cat #M1168 Cell Biologics). Cells were cultured on gelatin (Cell biologics) coated tissue culture dishes. Murine C2C12 cells (cat#CRL-1722 ATCC) were used at passages 2 through 10 and cultured in complete growth media (DMEM supplemented with 20%FBS (Atlanta Biologicals) and 1% Penicillin and streptomycin (Life Technologies)).

*TWEAK stimulation*. For stimulation experiments in MDEC cells were washed one time with 1 × PBS and then incubated overnight with low (10 ng ml^−1^) and high dose (150 ng ml^−1^) of mouse recombinant His tagged TWEAK (R&D), in Basal Endothelial Media (BM) (Cell Biologics) supplemented with 5% fetal bovine serum.

For stimulation in murine C2C12 cells, cells were washed one time with 1 × PBS and then incubated with low (10 ng ml^−1^) and high dose (100 ng ml^−1^) of mouse recombinant TWEAK for 2 h in differentiating media (DM) (that is, DMEM (ATCC) supplemented with 2% Horse serum(ATCC) and antibiotics). After exposure to TWEAK at the indicated doses and times, whole-cell lysates were subjected to immunoblotting as described below.

For experiments shown in Fig. 6c, mouse recombinant TWEAK (untagged) was obtained from Kingfisher Biotech.

*Macrophage supernatant experiments*. MDEC were plated in six-well plates (Falcon) at a concentration of 2.5 × 10^5^ cells ml^−1^ and incubated overnight in CGM. The next day media was aspirated and cells were treated with 7-day m-CSF-treated BMM supernatants obtained from WT and CD163^−/−^ differentiated BMMs. Each well was treated with WT supernatant or CD163 supernatant. Treatment lasted 3 h. Cells were harvested for immunoblotting using techniques and antibodies described below.

*Cell proliferation assays*. C2C12 cells were seeded at 2 × 10^3^ cells per well in GM in a 96-well plate (Falcon) and incubated overnight at 37 °C. The next day, cells were washed once with 1 × PBS and treated overnight with and without TWEAK (cat#1237 R&D Systems) (100 ng ml^−1^) in DM. Cells were then pulsed with 10 μM BrdU and incubated thereafter for 2 h. Media was removed and cells were fixed, and processed per manufacturer protocol. Cellular proliferation was determined immunochemically by detection of BrdU incorporated into newly synthesized DNA into cells which is directly proportional to the magnitude of colour development by using Calbiochem BrdU Cell Proliferation assay kit. Absorbance was measured at wavelength of 450 nm. Results for each experiment were expressed as fold change compared with the negative control, and the fold changes for three independent experiments were then averaged.

MDEC were seeded at 4 × 10^3^ cells per well and incubated in CGM in a 96-well plate and incubated overnight at 37 °C. Next day, Cells were washed once with 1 × PBS medium and replaced with BM supplemented with 5% FBS. Cells were treated or not treated with TWEAK (150 ng ml^−1^) overnight before pulsing with 10 μM BrdU. Cellular proliferation was determined by measurement of BrdU using Calbiochem BrdU Cell Proliferation assay as described above.

*Proliferation assay and siRNA transfection*. C2C12 cells were seeded at a concentration of 1 × 10^4^cells per well in GM on day 0. On day 1 the cells were transfected with scrambled Stealth RNAi siRNA duplex (36% GC) (negative) (proprietary sequence) or stealth P65siRNA GCAGAAAGAAGACAUUGAGGUGUAU, or stealth RBPJ siRNA CCAUUACGGGCAGACUGUCAAGCUU, (Life Technologies) using Lipofectamine 3000 (Life Technologies) as per manufacturer's protocol. Transfection was preformed in Optimem (Life Technologies).

Transfection complexes were removed after 6 h and replaced with GM. Twenty-four hours after transfection, that is, day 2 the media was aspirated, cells were washed 1 × PBS followed by addition of DM and treated or not treated with 100 ng ml^−1^ TWEAK recombinant protein overnight. Forty-eight hours post transfection, transfected cells were pulsed with 10 μM BrdU and incubated for 2 h. Cellular proliferation was determined as described above.

MDEC were seeded 1 × 10^5^ cells per ml and incubated in regular growth medium in a 96-well plate and incubated overnight at 37 °C. Next day cells were transfected with scrambled stealth siRNA or stealth P65 siRNA and RBPJ siRNA (sequences provided above) using Lipofectamine 3000 (Life Technologies) and Optimem. Transfection complexes were removed after 6 h and replaced with CGM. 24 h post transfection, media was aspirated and the cells were washed with 1 × PBS, followed by addition of 5% FBS in basal medium to the cells and treated or not treated with 150 ng ml^−1^ TWEAK recombinant protein for overnight. 48 h post transfection cells were then pulsed with 10 μM BrdU and incubated for 6 h. Cell proliferation was assayed as described above.

All siRNA sequences were validated in MDEC using western blots demonstrating >80% knockdown (Supplementary Fig. 16).

### Western blotting

Whole-cell lysates were prepared by resuspending the cell pellet in ice-cold RIPA lysis buffer supplemented with Protease inhibitors (Roche) and 10 mm PMSF and incubated at 4 °C for 20 min. Lysates were then clarified by centrifugation at 4 °C for 15 min. Protein concentrations were estimated using the BCA Protein Assay Kit (Pierce). Equal amounts of protein extract (∼100 ug) were subjected to SDS-polyacrylamide gel electrophoresis and electrotransferred onto PVDF membranes Blocking was performed using 1 × Casein blocking buffer in 1 × TBST. Membranes were then probed with primary antibodies diluted in 5% bovine serum albumin in 1 × TBST, and incubations proceeded O/N at 4 °C. Primary antibodies used were as mentioned above. The membranes were then washed three times in TBST for 5 min each and then incubated in TBST/1 × Casein containing a 1:1,000 dilution of horse radish peroxidase-conjugated goat anti-rabbit IgG- secondary antibodies, for 1 h at room temperature. The membranes were again subjected to three washes in TBST for 5 min each times. HRP was detected using the SuperSignal chemiluminescence reagent substrate (Pierce) and signal was visualized on the Biorad Chemdoc system. All experiments were performed at least three times. Full images of immunoblots are shown in Supplementary Figs 17–23.

### Analysis of Notch activation after TWEAK stimulation following p65 knockdown

MDEC were seeded at 1 × 10^6^ per 100 mm dish cultured at 37 °C in CGM overnight. C2C12 cells were seeded at 4 × 10^5^ per 100 mm dish cultured at 37 °C in GM overnight. Both C2C12 and MDEC were transfected with scrambled stealth RNAi siRNA duplex (36% GC) (negative) (proprietary sequence) and stealth P65siRNA GCAGAAAGAAGACAUUGAGGUGUAU using Lipofectamine 3000 and Optimem. Transfection complexes were left for 6 h before removal and replaced with CGM/GM. The transfected MDEC were then treated the next day with 5%FBS in BM and nothing or 150 ng ml^−1^ TWEAK for 24 h. The cells were harvested for western blot 48 h after transfection. The transfected C2C12 cells were treated 48 h post transfection with DM and with TWEAK (100 ng ml^−1^) or nothing for 2 h. Immunoblotting was then performed on whole-cell lysates as described above.

### ELISA

TWEAK concentrations were measured in plasma obtained from both genotypes of mice without HLI, 3, 14 and 28 days after HLI using commercial mouse TWEAK ELISA (cat#MBS721908, MyBiosource, *n*=5). Similarly sCD163 concentrations were measured by obtaining plasma from both genotypes of mice without HLI, 3, 14 and 28 days after HLI using commercial mouse sCD163 ELISA (cat#MBS725142, MyBiosource, *n*=5). All experiments were conducted according to manufacturer's directions.

### TWEAK binding ELISA

Recombinant mouse sCD163 (cat#7435 R&D Systems) and recombinant soluble Fn14/TWEAKR (cat#310-21Peprotech) were coated onto 96-well microtiter plate (Linbro MP Biomedicals) at a concentration of 1 μg ml^−1^ in bicarbonate/carbonate buffer (pH 9) overnight at 4 °C. VEGFR1 (cat#10136-H08H Life technologies) was used as a negative control. Wells were blocked with blocking buffer (Pierce) in phosphate buffered saline with 0.05% Tween 20 (PBST) for 1 h. PBST was used as wash buffer. Mouse soluble TWEAK (cat#1237 R&D Systems) was then added to the wells at 0.4 μg ml^−1^ for 30 min, at room temperature. The wells were washed three times using PBST. The binding of TWEAK to CD163/Fn14 was detected by ELISA using a biotinylated anti-mouse TWEAK antibody (cat#BAF1237 R&D Systems) diluted according to manufacturer's directions followed by PBST rinses repeated three times. To detect the biotinylated TWEAK antibody a HRP enzyme conjugated Streptavidin recombinant protein (cat#554066 BD Pharmingen) was added for 30 min at a 1:1,000 dilution. This step was again followed by triplicate PBST rinses. Bound HRP enzyme conjugate was detected using TMB (Pierce) as substrate for 15 min, and 2 N H_2_SO_4_ as the stop solution. Absorbance was measured using an automated plate reader at a wavelength of 450 nm.

To confirm coating of the proteins parallel ELISAs were performed using corresponding antibodies anti CD163 (M-96) (sc-33560 Santacruz) and anti FN14 (cat#4403 Cell Signaling) followed by an HRP-conjugated anti-rabbit secondary antibody. Coated proteins were detected using TMB as substrate as described above.

To further confirm specificity of binding of the detection antibody, nothing or recombinant mouse TWEAK was added to uncoated pre-blocked wells during the experiment followed by the addition of biotinylated anti-mouse TWEAK antibody. The HRP conjugate Streptavidin was then added as stated above. There was no significant difference in signal generated from these experiments. This ruled out non-specific binding of the detection antibody and of recombinant TWEAK.

To rule out that detection of TWEAK using an anti-TWEAK antibody might in some way be affected by TWEAK binding to CD163 or FN14, wells were coated with TWEAK (0.4 μg ml^−1^). CD163 (1 μg ml^−1^) and FN14 (1 μg ml^−1^), or nothing was added to the blocked TWEAK-coated wells for 1 h. Wells were washed as per protocol. Detection of TWEAK using anti-TWEAK antibody revealed no differences in optical density between groups suggesting that binding of TWEAK to either receptor does not influence the detection method.

For experiments showing dose-dependent TWEAK binding to recombinant mouse sCD163 and recombinant soluble FN14, individual wells were coated with CD163 (1 μg ml^−1^)and Fn14(1 μg ml^−1^). Wells were first blocked and increasing concentrations of untagged recombinant mouse TWEAK (Kingfisher Biotech) (0, 3, 10, 30, 100, 300 and 1,000 ng ml^−1^) were added to the wells for 30 min at room temperature followed by biotinylated anti-TWEAK and HRP-conjugated Streptavidin as described earlier.

### Serum immunoprecipitation

Pierce Co-Immunoprecipitation kit Cat# 26149 was used for the Co-Immunoprecipitation of the Tweak-CD163 complex from serum. All steps were performed as per kit protocol. Affinity purified anti-TWEAKpAb (ALX-210-926 Enzo Life Sciences) was cross-linked to AminoLink Plus coupling Resin. Normal Rabbit IgG (cat#sc-2027 Santacruz) was used instead of the TWEAK antibody as an isotype control. 2.2 ml of pooled mouse serum was mixed in a 1:1 ratio with 2 × PBS along with protease inhibitors (Roche), and then introduced to the Anti-TWEAK cross-linked beads after which the antibody–antigen complex was gently shaken overnight at 4 °C using an end over end rotator. Co-immunoprecipitated protein complexes were eluted with given elution buffer. Samples were mixed with 4 × sample buffer supplemented with 20 mm dithiothreitol and heated at 95 °C for 5 min followed by centrifugation at 13,000 r.p.m. for 2 min at 4 °C before applying onto a 4–15% TGX gel (Biorad) Membranes were probed using an anti CD163(M-130) (cat#bs2525R Bioss Antibodies) and anti TWEAK pAb (Enzo). Recombinant mCD163 (R&D) was used as positive control.

### Analysis of angiogenesis in the postnatal mouse retina

To analyze retinal vascular pattern and to preserve intact endothelial filopodia and sprouts at the angiogenic front, whole animal eyes were fixed in 4% paraformaldehyde 4 °C overnight. After washing with PBS, retinas were dissected and permeabilized in PBS, 1% BSA, and 0.5% Triton for 2 h at 4 °C. After blocking/permeabilization, retinas were incubated in FITC-conjugated isolectin B4 (Cat.#L2895, Sigma) for 24 h at 4 °C. For DLL4 detection, an antibody against DLL4 (cat# AF1389R&D Systems)was used. The following day, the retinas were washed in PBS before the retinas were partially cut in four quadrants to allow subsequent flat mounting with Vectashield mounting medium (Vector Labs, Burlingame, CA). All of the images shown are representative of the vascular phenotype observed in at least ten retinas from five pups. All quantifications were done with NIS-elements v3.0 (Nikon) on high-resolution confocal images. The ratio of the isolectin-B4-positive area to the total area was calculated and defined as a percentage. Vascular progression was measured by defining a straight line from the angiogenic front to the centre of the retina. The number of filopodial extensions was quantified at the retina angiogenic front. The total number of filopodia was normalized for a standard endothelial vessel length of 1 mm that was measured and defined according to published protocols^40^. Branchpoint is assessed as the number per 200 × 200 um field.

### Statistics

Analysis of equal variances was performed using an O'Brien test. Analysis of normality of continuous variables was performed using the Kolmogorov–Smirnov test. Normally and non-normally distributed data were analysed for significance by student's *t*-test or Mann–Whitney–Wilcoxon tests, respectively. For mouse experiments, we estimated that a sample size of five mice per group with an expected difference of roughly 40% in terms of end point with a standard deviation of 15% would provide >90% power to detect a differences between groups using a two-sided alpha of 0.05. For quantification of other studies presented, a minimum of 4 replicates were tested for each condition of experiment. The data are represented as mean and s.e.m. Comparisons between groups were achieved using a two-sided student's *t*-test. For multiple group comparisons, we utilized a one-way analysis of variance (ANOVA). If the variance ratio test (*F*-test) was significant, a more detailed *post hoc* analysis of differences between groups was made using a Tukey–Kramer honest significance difference test. For analysis of running distance, a two-way ANOVA was used to analyze differences. The *P* value for interaction between the two groups (mouse genotype and procedure (that is, HLI versus sham) was 0.03. Differences were deemed to be statistically significant for *P* values of <0.05. One animal in the running wheel experiment (Supplementary Fig. 2b) in CD163 HLI group was excluded when it was found his cage was not functioning properly. No other animals were excluded from the analyses.

## Additional information

**How to cite this article:** Akahori, H. *et al.* CD163 interacts with TWEAK to regulate tissue regeneration after ischaemic injury. *Nat. Commun.* 6:7792 doi: 10.1038/ncomms8792 (2015).

## Supplementary Material

Supplementary Information Supplementary Figures 1-23
