# Supplementary material for: CD163 interacts with TWEAK to regulate tissue regeneration after ischaemic injury
Source: Nat Commun. 2015 Aug 5;6:7792. doi: 10.1038/ncomms8792 (PMC4918310; doi:10.1038/ncomms8792)
Supplement: Supplementary Information — Supplementary Figures 1-23 [file ncomms8792-s1.pdf]

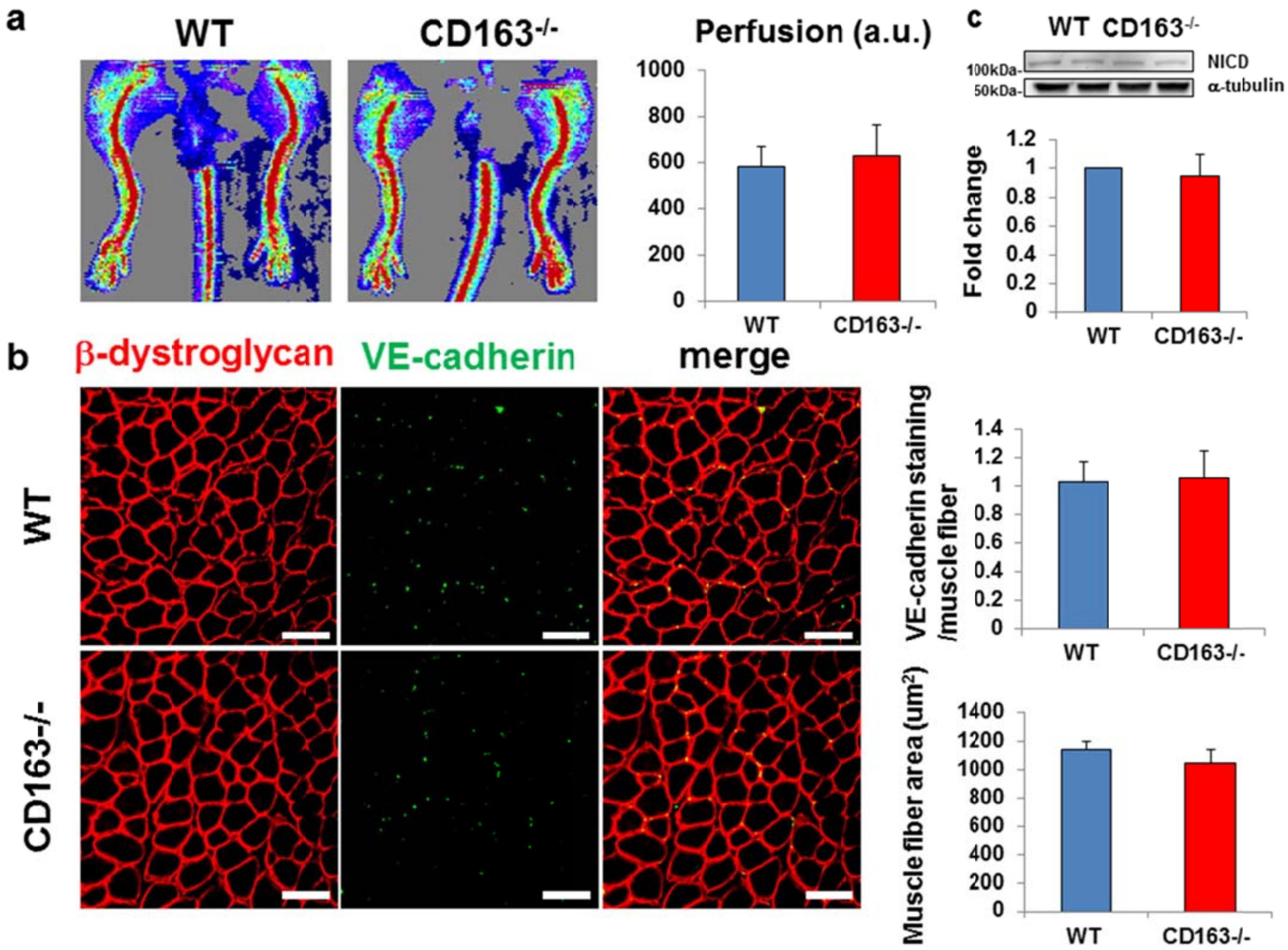

Supplementary Figure 1. CD163$^{-/-}$ mice exhibit a similar phenotype as WT mice in the absence of ischemic injury.

a, Laser Doppler analysis with perfusion quantitation at baseline (n=10 per group). **b,** Immunostaining of limb for VE-cadherin (green) and β-dystroglycan (red) (n=4 per group). The graphs (right) show the number of VE-cadherin positive cells/myofiber and the average muscle fiber area (n=5 per group). Scale bars indicate 100um. c. immunoblotting of skeletal muscle of naïve mice with densitometry quantitation for Notch intracellular domain (NICD) below (n=4 per group). All bars show mean ± s.e.m. Comparisons between groups were achieved using a two-sided student's t-test.

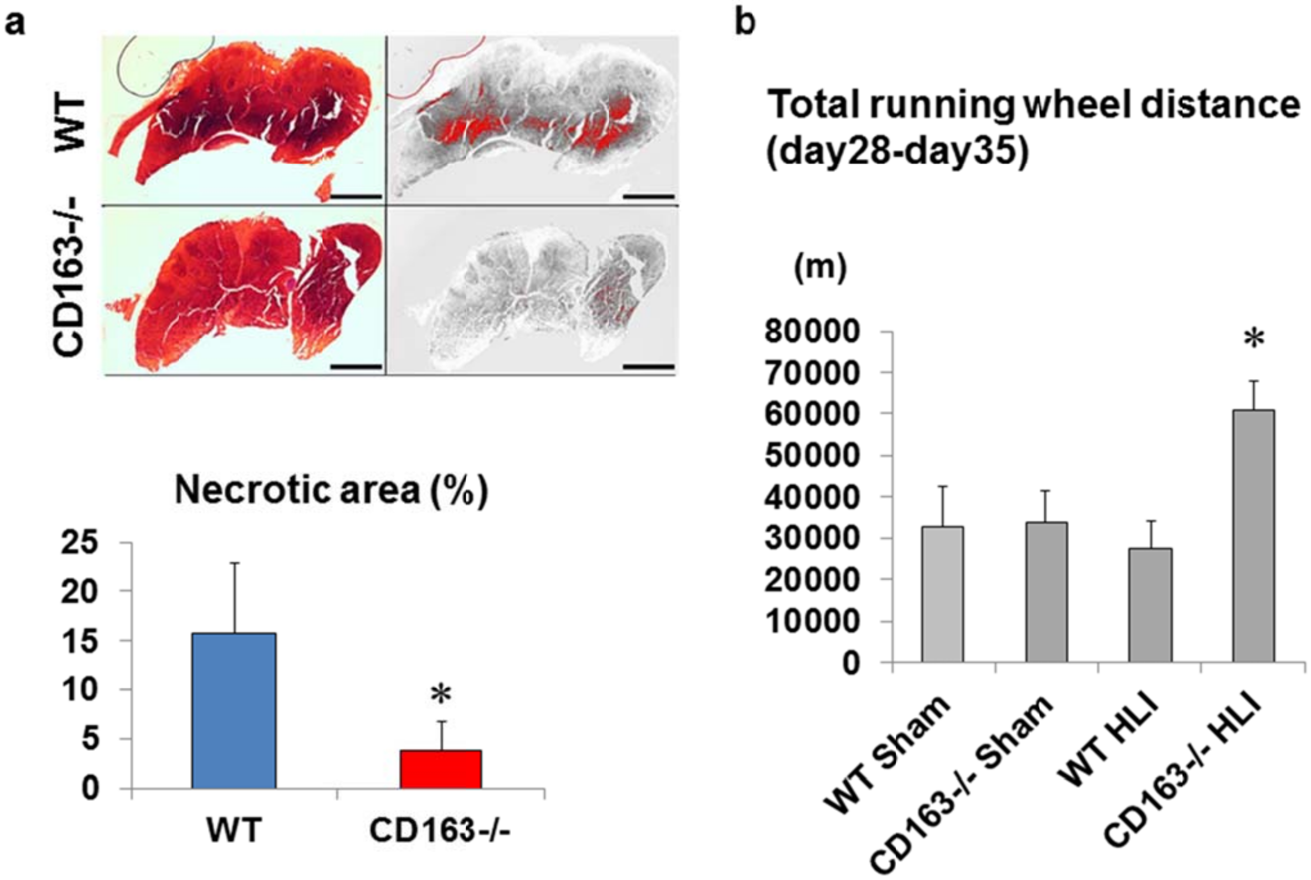

Supplementary Figure 2. Necrotic area 14 days after femoral ligation and running wheel distance 28 days after femoral ligation in WT and CD163$^{-/-}$ mice.

a, Haematoxylin and eosin staining of ischemic limb 14 days after femoral ligation. Area of ischemic necrosis is shown in red in pictures on right. Scale bars indicate 1mm. The graph shows quantitation of haematoxylin and eosin stained section of ischemic limbs (n=5 per group). **b,** Cumulative treadmill running test on days 28 through 35 after femoral ligation (n=10 per group). All bars show mean ± s.e.m. *, $p<0.05$ versus WT in a; versus all other groups in b. Comparisons between groups were achieved using a two-sided student's t-test. For analysis of running distance, a two-way ANOVA was used to analyze differences.

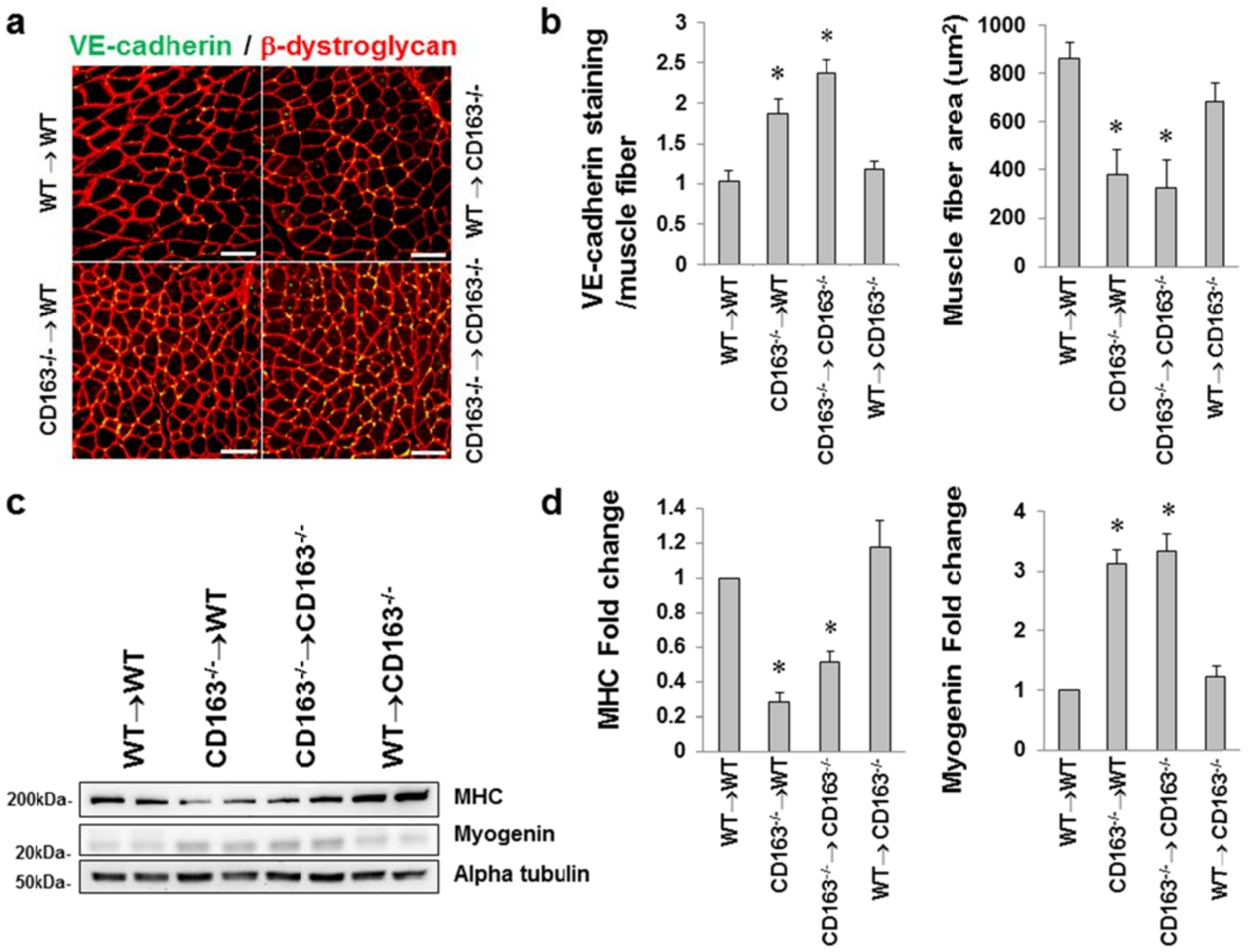

Supplementary Figure 3. Myeloid CD163 deletion is responsible for the phenotype of CD163-/- mice after femoral ligation.

a, Immunostaining for VE-cadherin (green) and β-dystroglycan (red) in 28 day ischemic limbs after bone marrow transplantation. WT bone marrow into WT and CD163-/- recipient mice (referred to as WT→WT and WT→CD163-/-, respectively); CD163-/- bone marrow into WT and CD163-/- recipient mice (referred to as CD163-/-→WT and CD163-/-→CD163-/-, respectively). Scale bars indicate 100um. **b**, Quantitation of VE-cadherin staining per muscle fiber and muscle fiber area in ischemic limb 28 days after bone marrow transplantation (n=5 per group). **c**, Immunoblotting of 28 day ischemic limbs after bone marrow transplantation for myosin heavy chain (MHC) and myogenin. **d**, Quantitation of protein expression (n=5 per group). All bars show mean ± s.e.m. *, $p<0.05$ versus WT→WT and WT→CD163-/-. For multiple group comparisons, we utilized a one-way ANOVA. If the variance ratio test (F-test) was significant, a more detailed post hoc analysis of differences between groups was made using a Tukey-Kramer honest significance difference test.

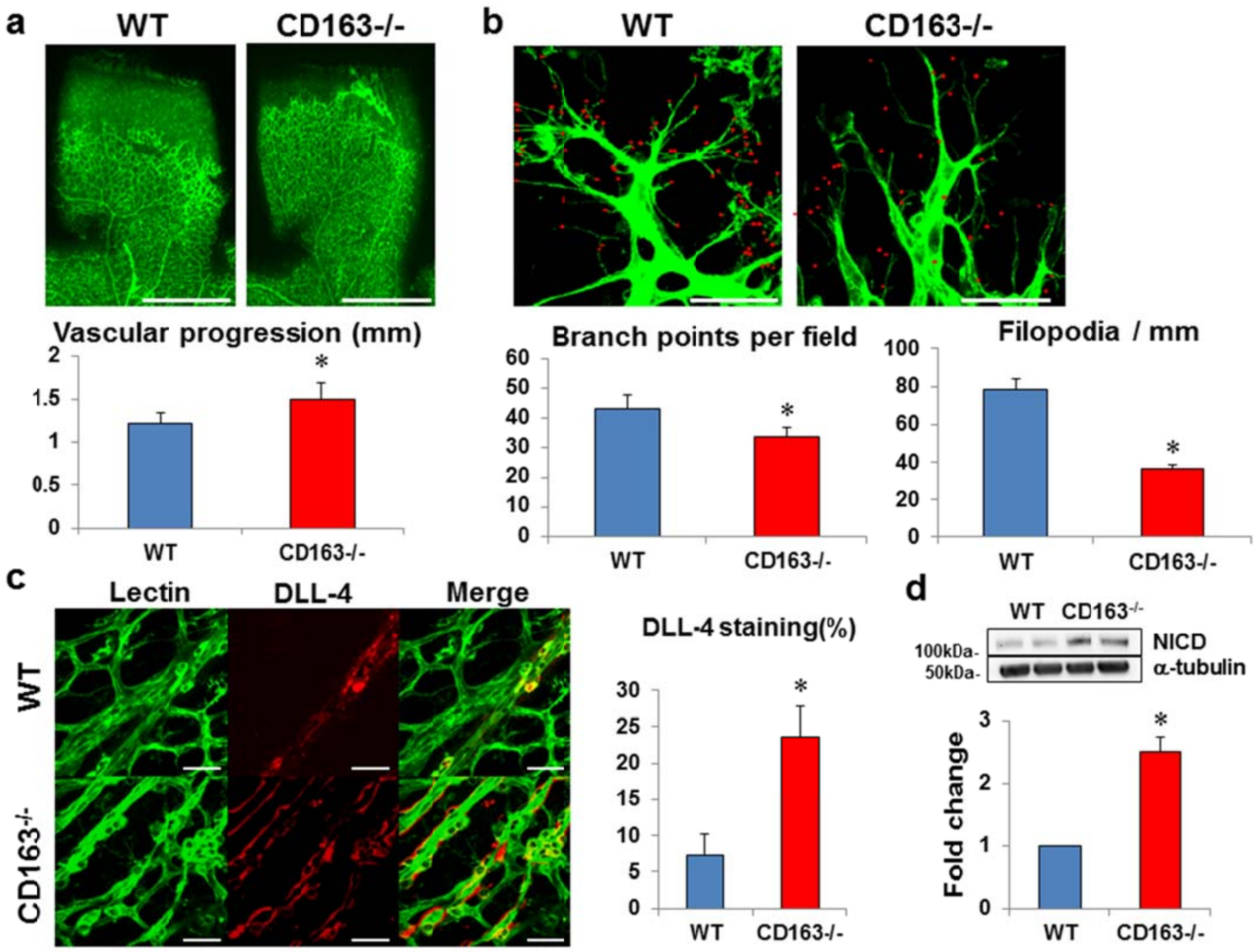

Supplementary Figure 4. CD163 deficient mice demonstrate Notch activation during retinal vascular patterning.

a, Representative confocal images of isolectin stained P6 retinas. Scale bars indicate 500um. The graph shows quantitation of vascular progression (n=10 per group). **b**, Representative high power confocal images of isolectin stained angiogenic front in P6 retinas. Scale bars indicate 50um. The bar graphs show the extent of vascular progression, the number of branch point per field, and filopodia per length of vascular front in P6 retinas (n=10 per group). **c**, Immunostaining of WT and $CD163^{-/-}$ P6 retinas for isolectin (green) and DLL-4 (red). The graph (right) shows quantification of percent of DLL-4 staining in P6 retinas (n=5 per each group). Scale bars indicate 100um. **d**, Immunoblotting of P6 whole retinas for Notch intracellular domain (NICD) (n=5 per group). All bars show mean ± s.e.m. *, $p<0.05$ versus WT. Comparisons between two groups were achieved using a two-sided student's t-test.

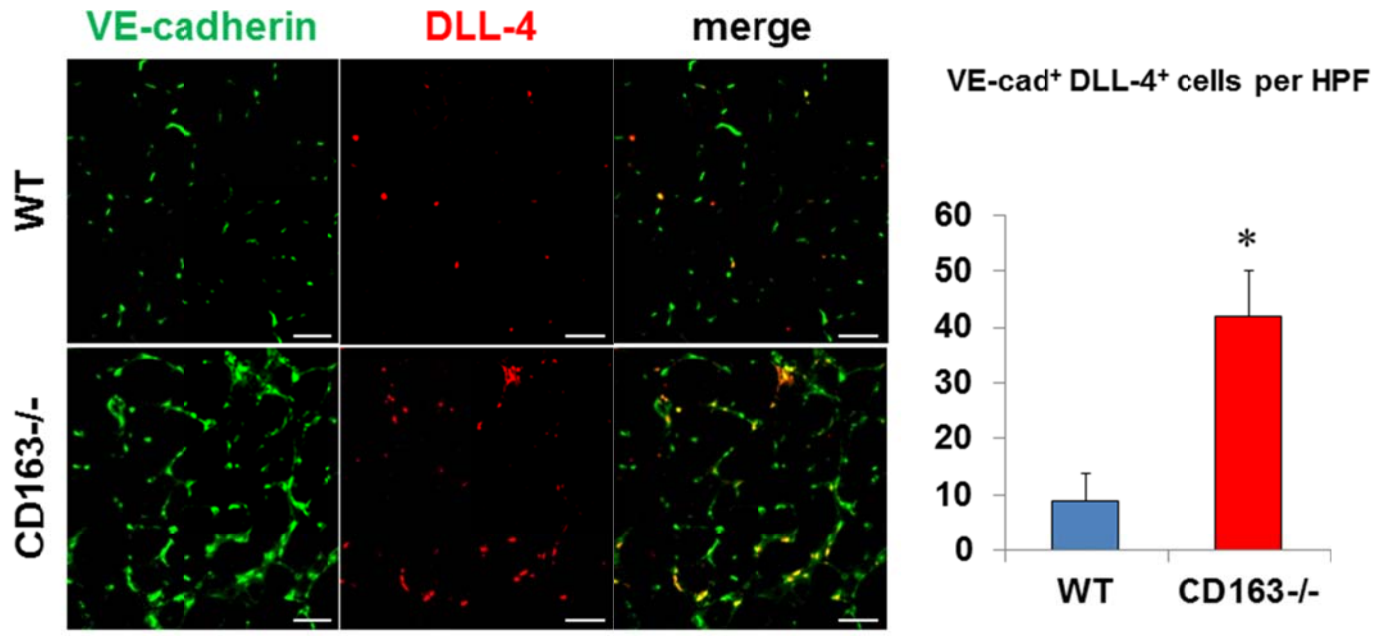

Supplementary Figure 5. CD163 deficient mice demonstrate increased in DLL-4 positive endothelial cell after femoral ligation.

Immunostaining of ischemic limb for VE-cadherin (green) and DLL-4 (red) at 14 days after femoral ligation. The graph shows the number of double positive cells per high power filed (HPF) (n=5 per each group). Scale bars indicate 20um. All bars show mean ± s.e.m. *, $p<0.05$ versus WT. Comparisons between two groups were achieved using a two-sided student's t-test.

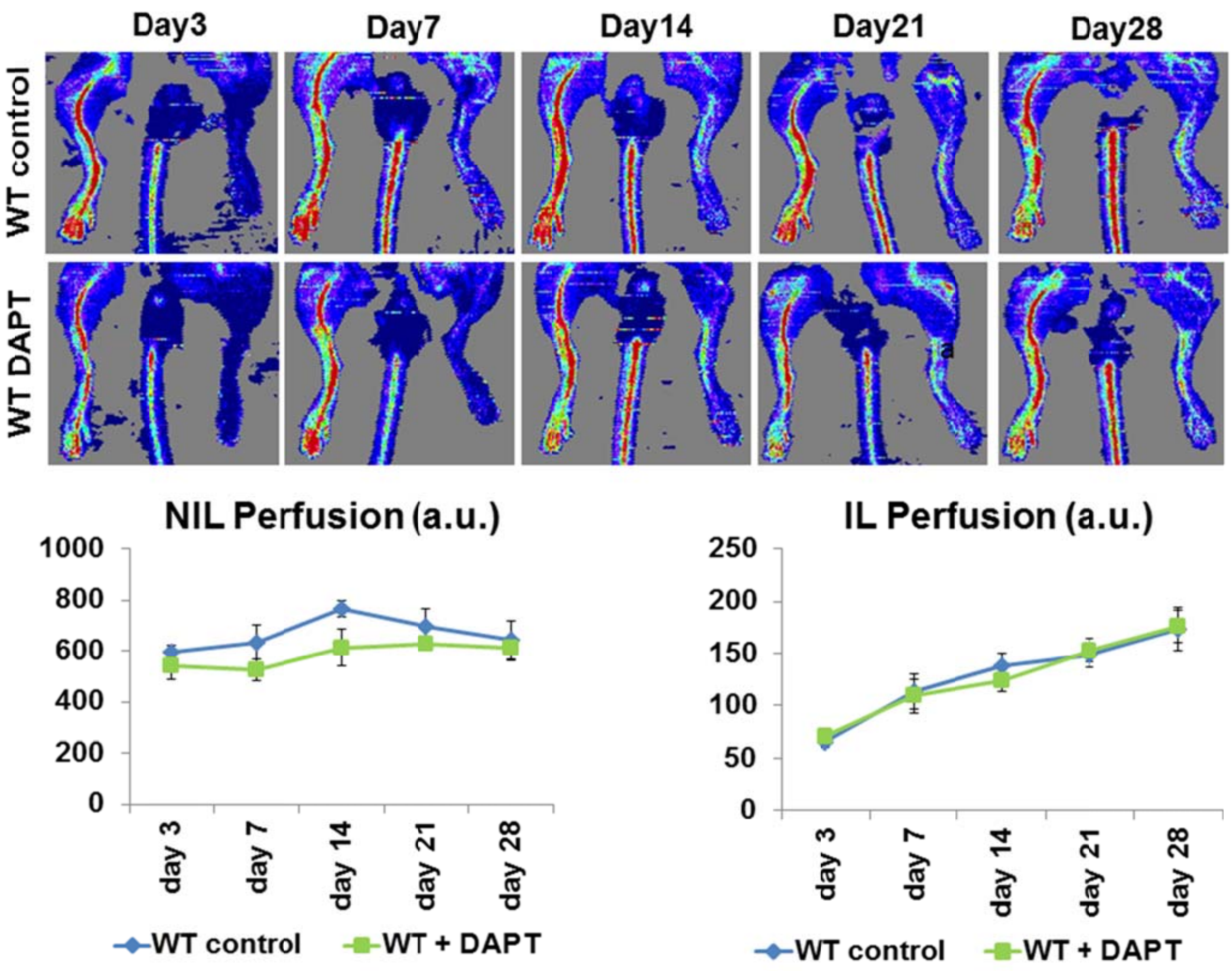

Supplementary Figure 6. DAPT treatment has no effect on blood flow recovery in WT mice.
Laser Doppler analysis of WT mice with perfusion quantitation 3, 7, 14, 21, and 28 days after femoral ligation with or without administration of Notch inhibitor DAPT (n=5 per group). IL=ischemic limb. NIL=non-ischemic limb. All bars show mean ± s.e.m. Comparisons between groups were achieved using a two-sided student's t-test.

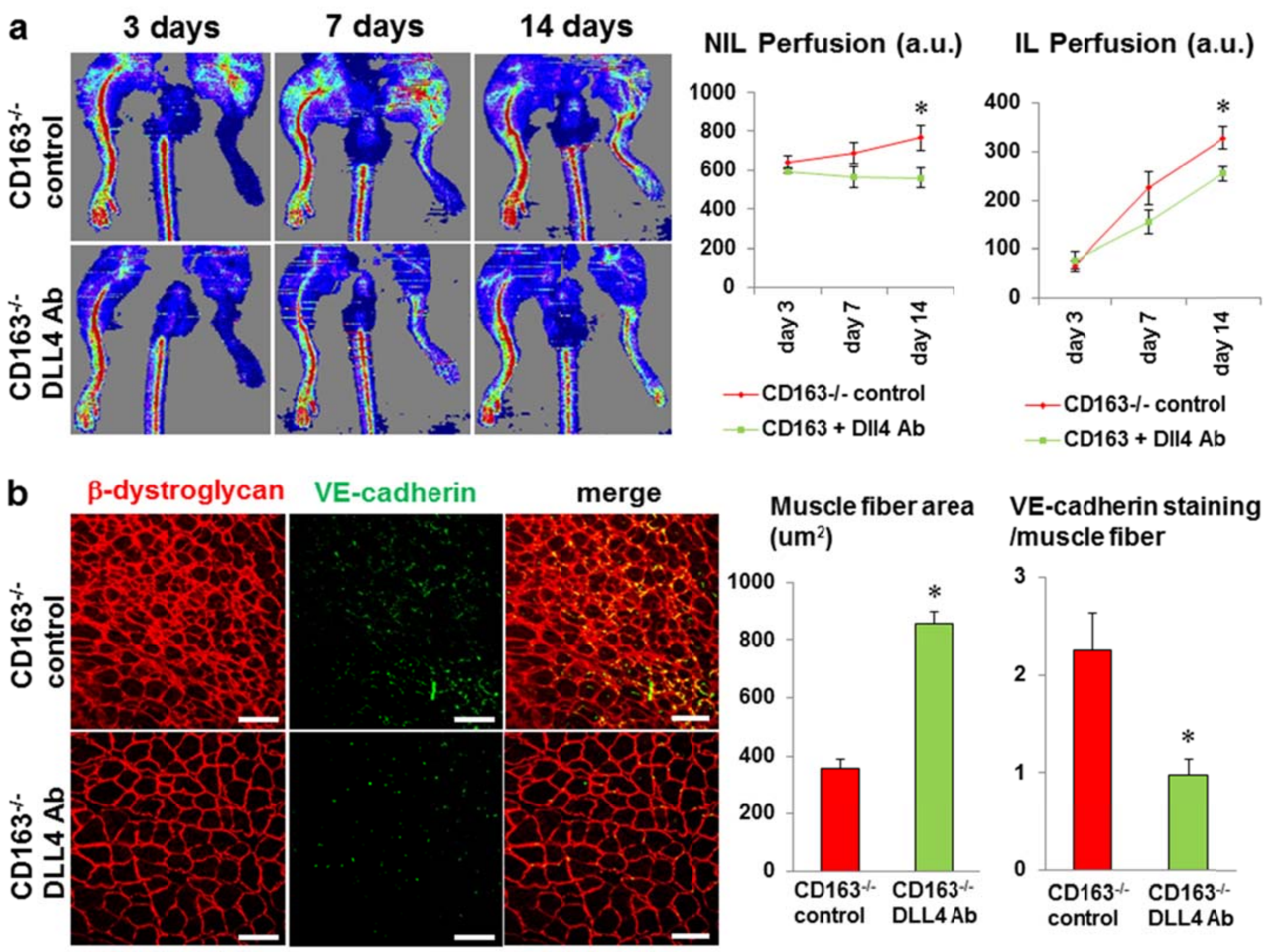

Supplementary Figure 7. DLL4 blocking antibody inhibits blood flow recovery and muscle morphologic responses of CD163$^{-/-}$ mice to limb ischemia.

a, Laser Doppler analysis of CD163$^{-/-}$ mice with perfusion quantitation 3, 7, and 14 days after femoral ligation with or without administration of a isotype control (control) or DLL4 blocking antibody (DLL4 Ab) (n=5 per group). **b,** Immunostaining of ischemic limb 14 days after femoral ligation for VE-cadherin (green) and β-dystroglycan (red). Scale bars indicate 100um. The graph shows quantitation of VE-cadherin staining per muscle fiber and muscle fiber area (n=5 per group). IL=ischemic limb. NIL=non-ischemic limb. All bars show mean ± s.e.m. *p<0.05 versus CD163$^{-/-}$ control. Comparisons between groups were achieved using a two-sided student's t-test.

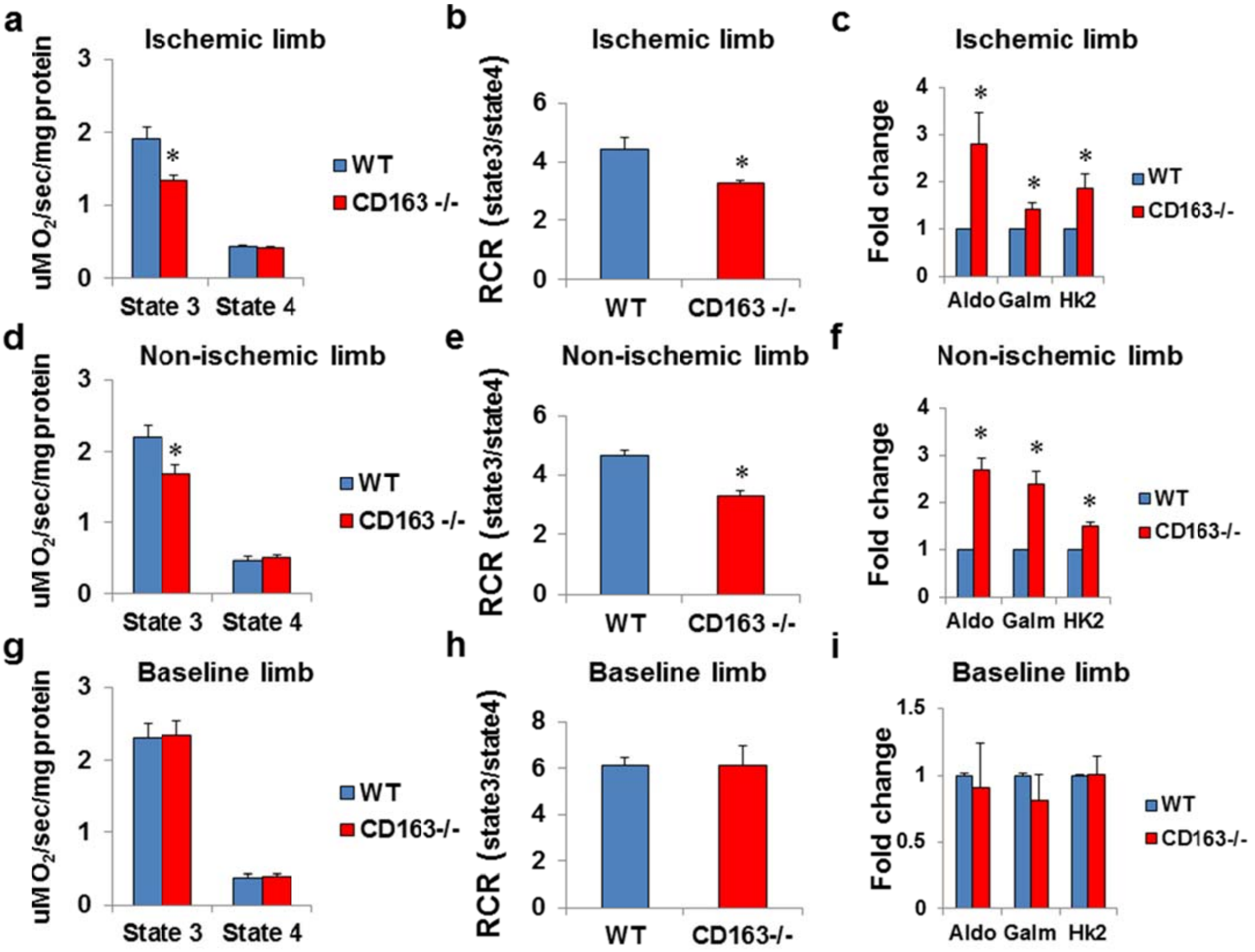

Supplementary Figure 8. CD163 deficiency affects mitochondrial function after ischemic injury.

a, d, g, Mitochondrial respiration from selected tissue skeletal muscle samples (ischemic limb 14 days after femoral ligation, non-ischemic limb 14 days after femoral ligation, and baseline limb, respectively). **b, e, h,** The respiratory control ratio (RCR, State 3/State 4 respiration) of tissue sample (ischemic limb 14 days after femoral ligation, non-ischemic limb 14 days after femoral ligation, and baseline limb respectively). n=5 per group. c,f,i, Quantitative PCR analysis of selected tissue skeletal muscle samples (ischemic limb 14 days after femoral ligation, non-ischemic limb 14 days after femoral ligation, and baseline limb, respectively). All bars show mean ± s.e.m. *, $p<0.05$ versus WT. Comparisons between two groups were achieved using a two-sided student's t-test.

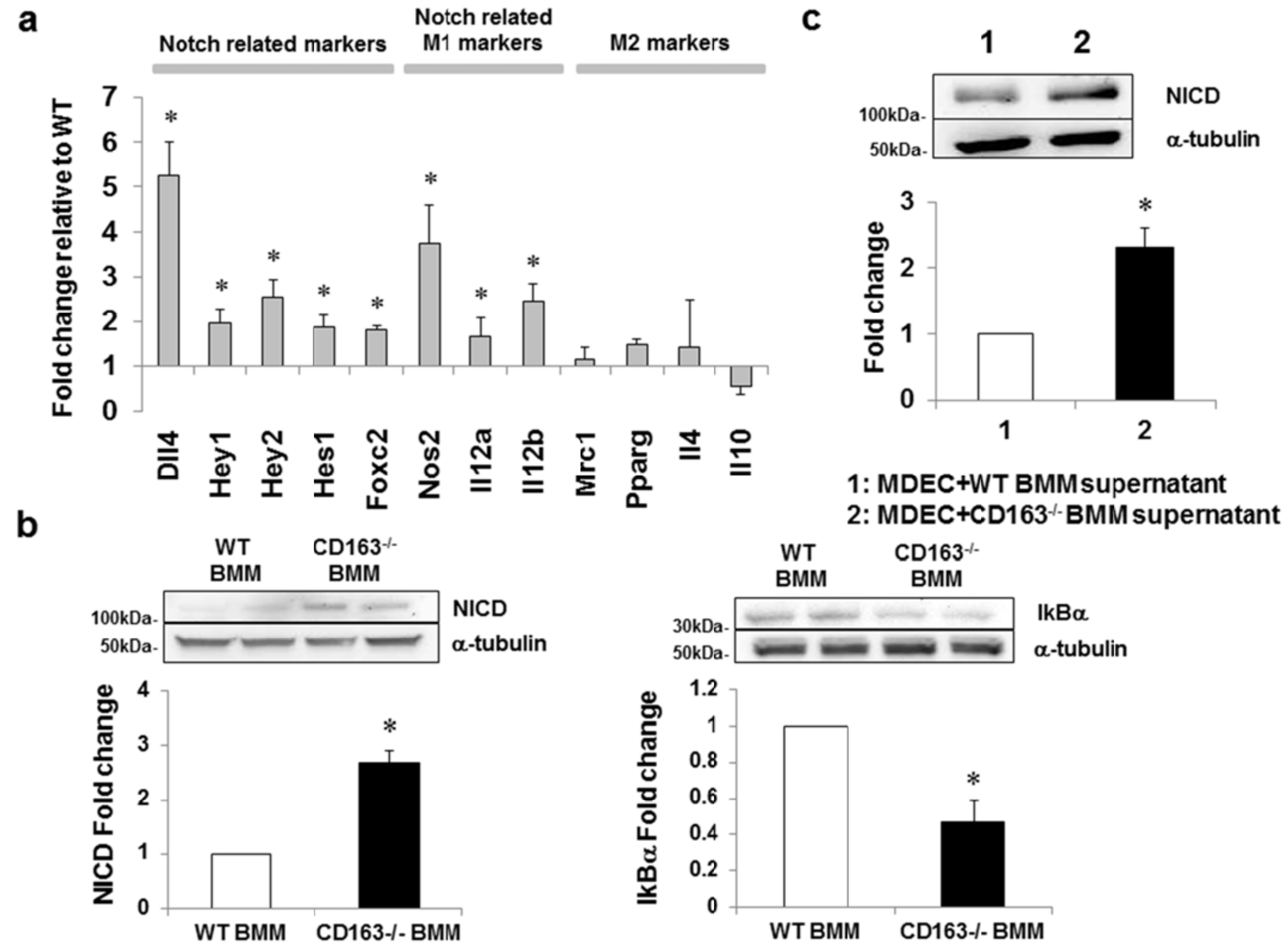

Supplementary Figure 9. CD163$^{-/-}$ bone marrow-derived macrophages (BMM) activate Notch signaling in a paracrine fashion.

a, BMM from WT and CD163$^{-/-}$ mice were differentiated in media supplemented with monocyte stimulating factor (M-CSF) for 7 days and macrophages were harvested for RNA analysis of selected transcripts by QPCR (n=4 per group). Bar graph shows fold change in transcript in CD163$^{-/-}$ mice relative to WT. **b**, Immunoblotting of BMM for Notch intracellular domain (NICD) and IkBα (n=4 per group). Graphs (bottom) show quantitation of densitometry. **c**, Immunoblotting of endothelial cells cultured with supernatants from WT and CD163$^{-/-}$ BMM for 3 hours (n=4 per group). Graph shows quantitation of densitometry. All bars show mean ± s.e.m. *, $p<0.05$ versus WT BMM group. Comparisons between groups were achieved using a two-sided student's t-test.

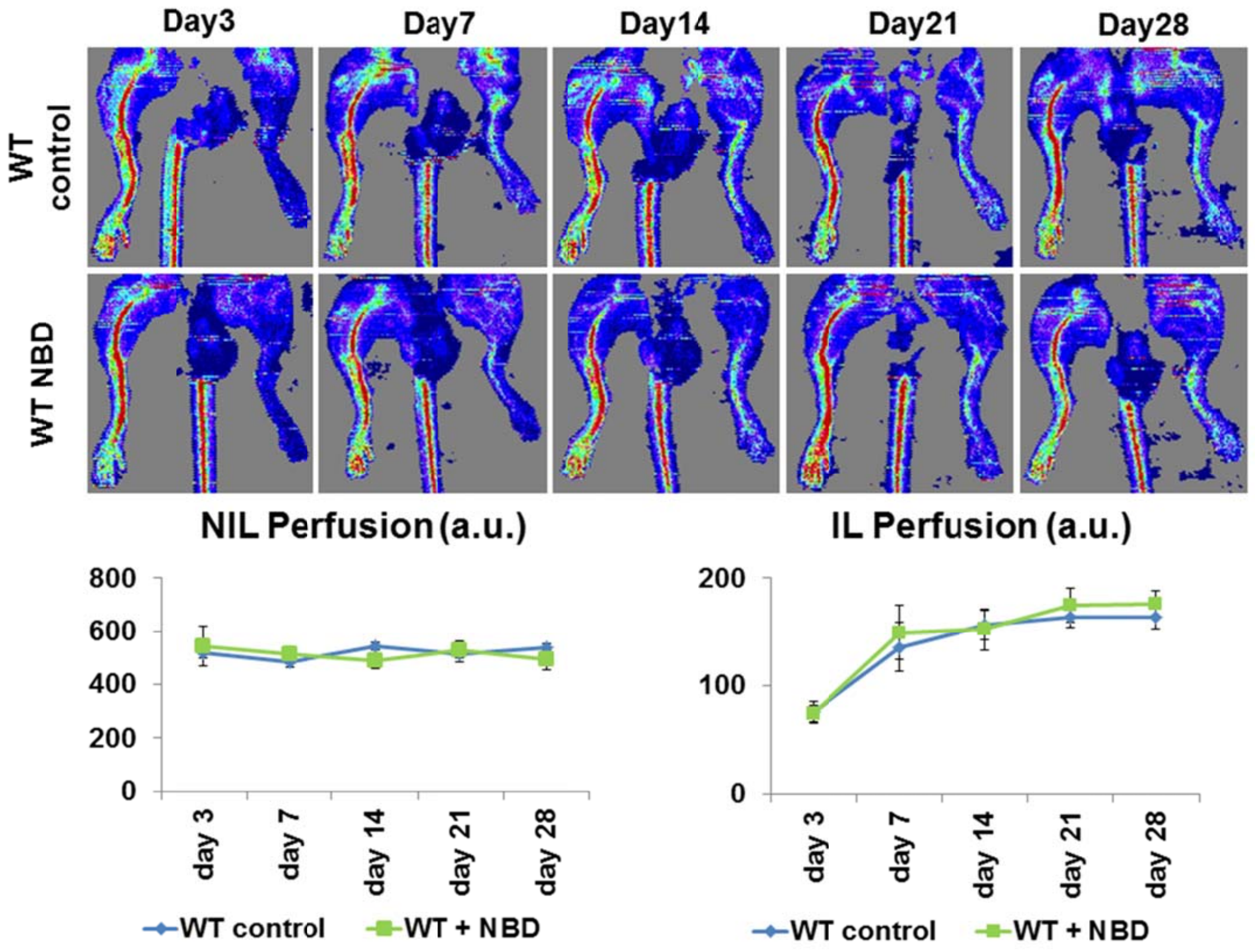

Supplementary Figure 10. NBD treatment has no effect on blood flow recovery in WT mice.
Laser Doppler analysis of WT mice with perfusion quantitation 3, 7, 14, 21, and 28 days after femoral ligation with or without administration of NFκB inhibitor NBD (n=5 per group). IL=ischemic limb. NIL=non-ischemic limb. All bars show mean ± s.e.m. Comparisons between groups were achieved using a two-sided student's t-test.

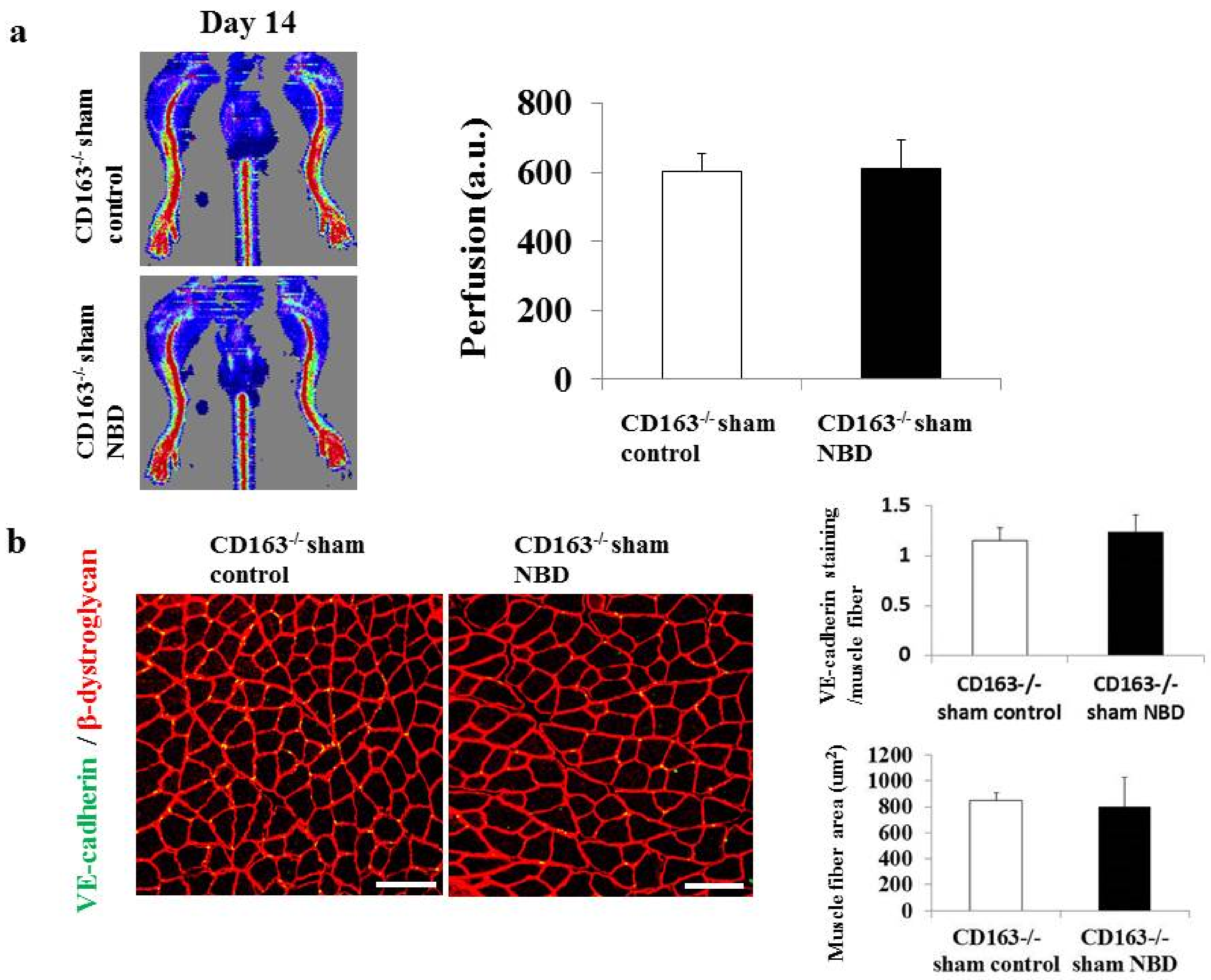

Supplementary Figure 11. NBD has no effect on blood flow and muscle morphology in CD163$^{-/-}$ mice without ischemic injury.

a, Laser Doppler analysis of CD163$^{-/-}$ mice with or without 14 days administration of control peptide or NFκB inhibitor NBD (n=5 per group). **b,** Immunostaining of CD163$^{-/-}$ limb after 14 days administration of control peptide or NBD for VE-cadherin (green) and β-dystroglycan (red). Scale bars indicate 100um. The graph shows quantitation of VE-cadherin staining per muscle fiber and muscle fiber area (n=5 per group). All bars show mean ± s.e.m. Comparisons between groups were achieved using a two-sided student's t-test.

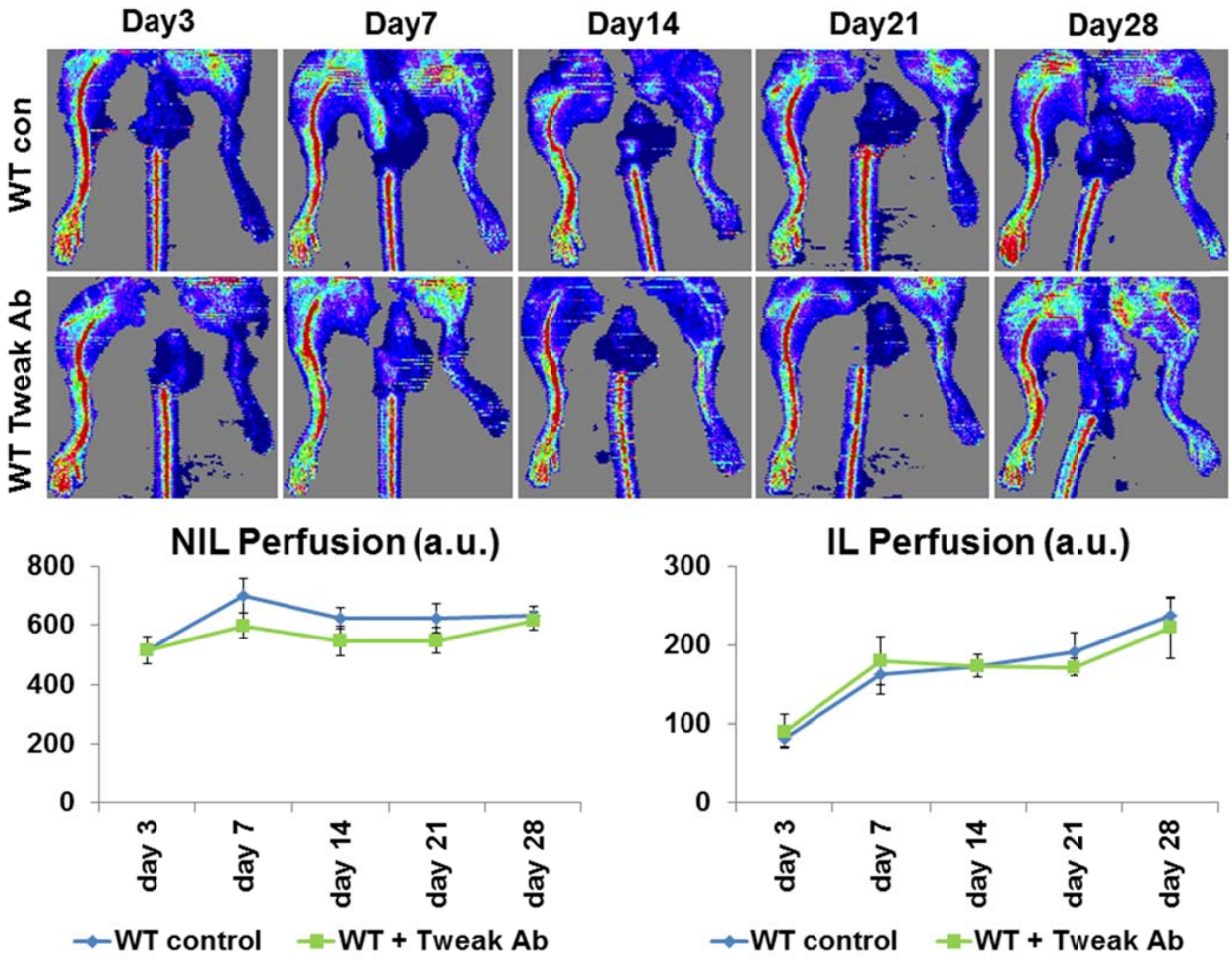

Supplementary Figure 12. TWEAK blocking Ab treatment has no effect on blood flow recovery in WT mice. Laser Doppler analysis of WT mice with perfusion quantitation 3, 7, 14, 21, and 28 days after femoral ligation with of an isotype control or TWEAK blocking Ab (n=5 per group). IL=ischemic limb. NIL=non-ischemic limb. All bars show mean ± s.e.m. Comparisons between groups were achieved using a two-sided student's t-test.

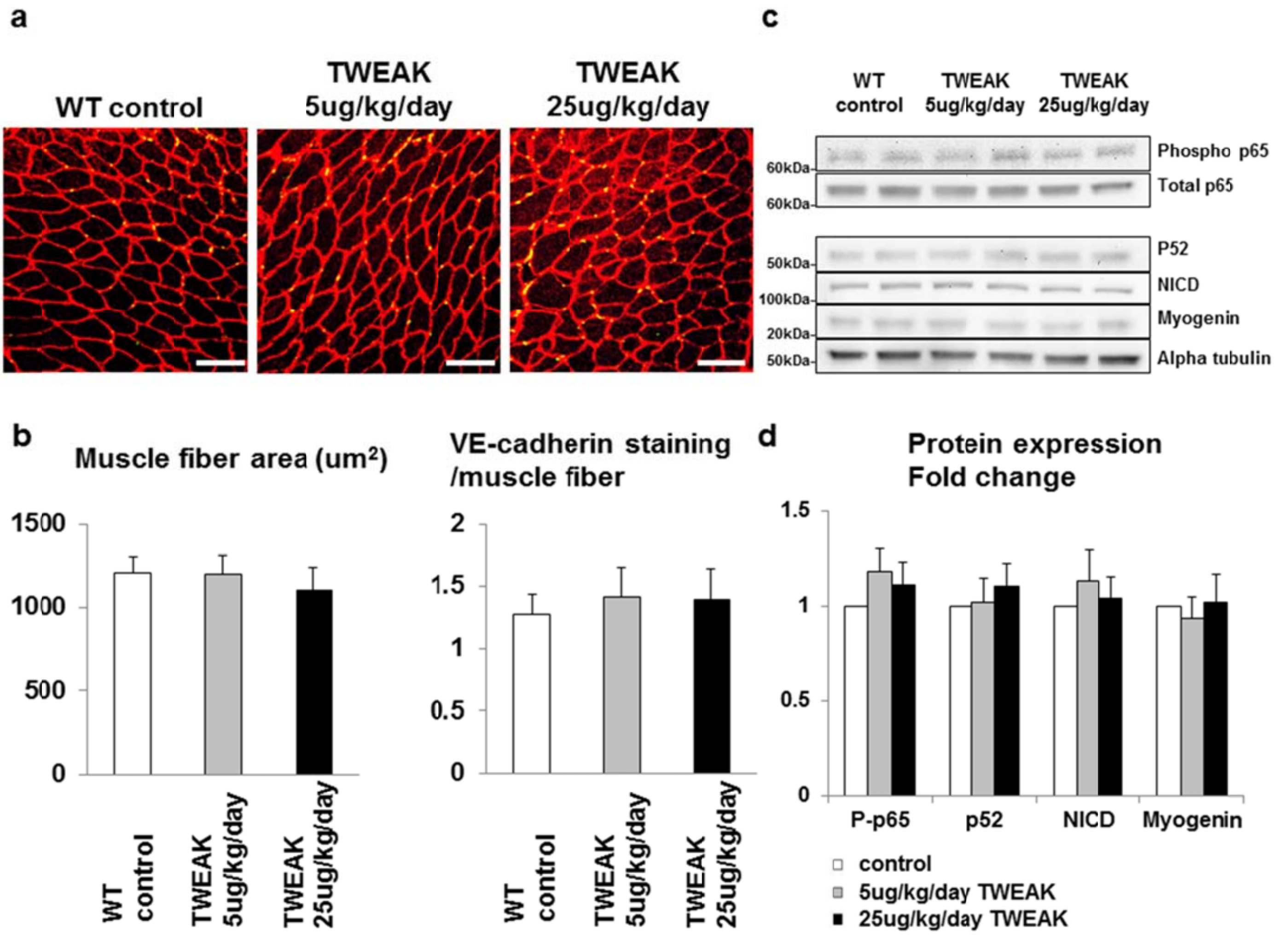

Supplementary Figure 13. TWEAK has no effect on skeletal muscle morphology, vascularity, and molecular signaling.

a, Immunostaining of WT limb with or without 14 day administration of TWEAK (5ug/kg/day or 25ug/kg/day) for VE-cadherin (green) and β-dystroglycan (red). Scale bars indicate 100um. **b,** Quantitation of VE-cadherin staining per muscle fiber and muscle fiber area (n=5 per group). **c,** Immunoblotting of WT$^{-/-}$ limb with or without 14 day administration of TWEAK (5ug/kg/day or 25ug/kg/day). **d,** Quantitation of protein expression for phosphorylated and total p65, p52, Notch intracellular domain (NICD), and Myogenin (n=5 per group). All bars show mean ± s.e.m. For multiple group comparisons, we utilized a one-way ANOVA. If the variance ratio test (F-test) was significant, a more detailed post hoc analysis of differences between groups was made using a Tukey-Kramer honest significance difference test.

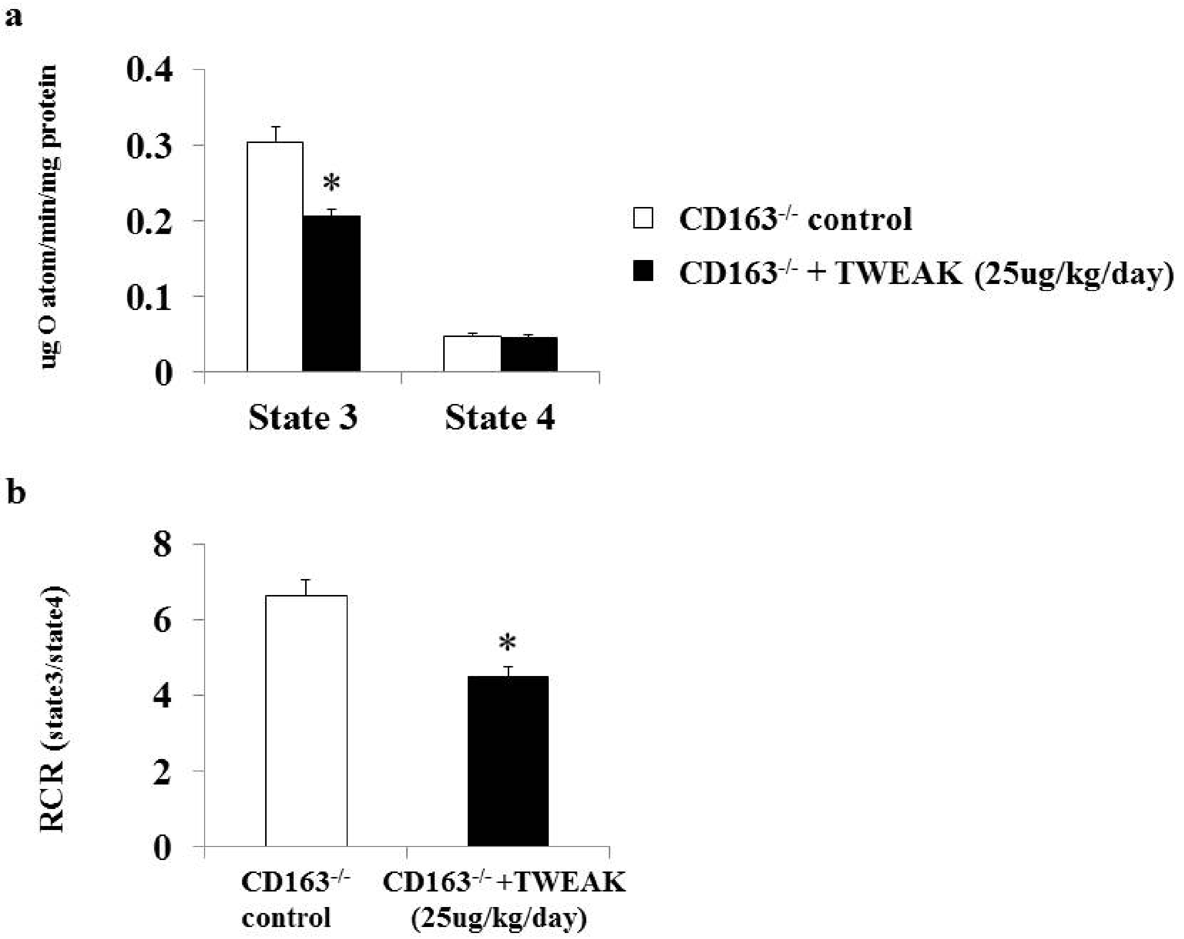

Supplementary Figure 14. High dose TWEAK affects muscle mitochondrial function in CD163-/- mice.
a, Mitochondrial respiration from CD163$^{-/-}$ limb with or without administration of TWEAK (25ug/kg/day). **b**, The respiratory control ratio (RCR; State 3/State 4 respiration) of CD163$^{-/-}$ limb with or without administration of TWEAK (25ug/kg/day). n=5 per group. All bars show mean ± s.e.m. *, $p<0.05$ versus CD163$^{-/-}$ control. Comparisons between groups were achieved using a two-sided student's t-test.

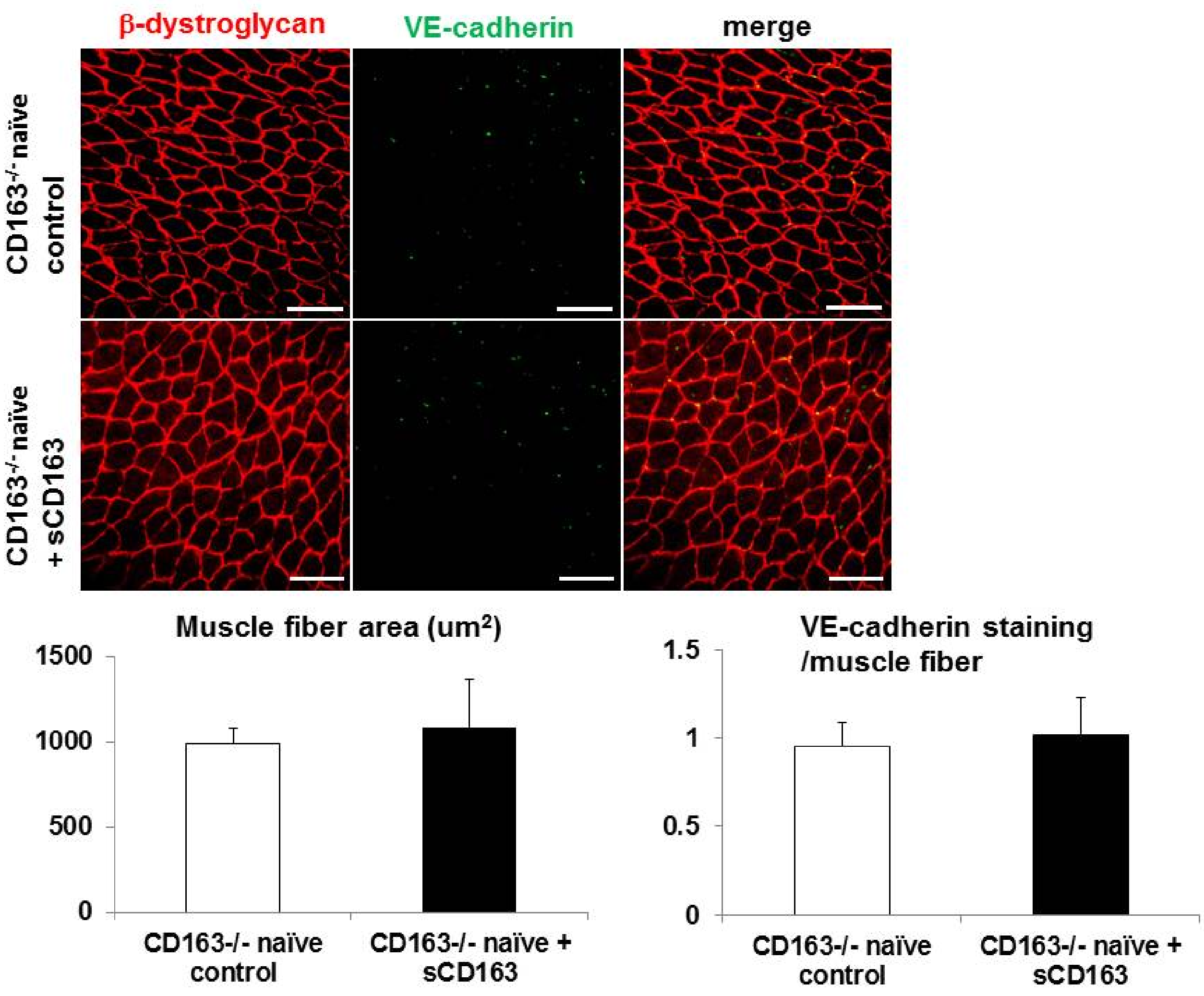

Supplementary Figure 15. sCD163 has no effect on vascularity and muscle morphology in CD163-/- mice without ischemic injury.

Immunostaining of CD163$^{-/-}$ limb after 14 days administration of sCD163 (25ug/kg/day) for VE-cadherin (green) and β-dystroglycan (red). Scale bars indicate 100um. The graph shows quantitation of VE-cadherin staining per muscle fiber and muscle fiber area (n=5 per group). All bars show mean ± s.e.m. Comparisons between groups were achieved using a two-sided student's t-test.

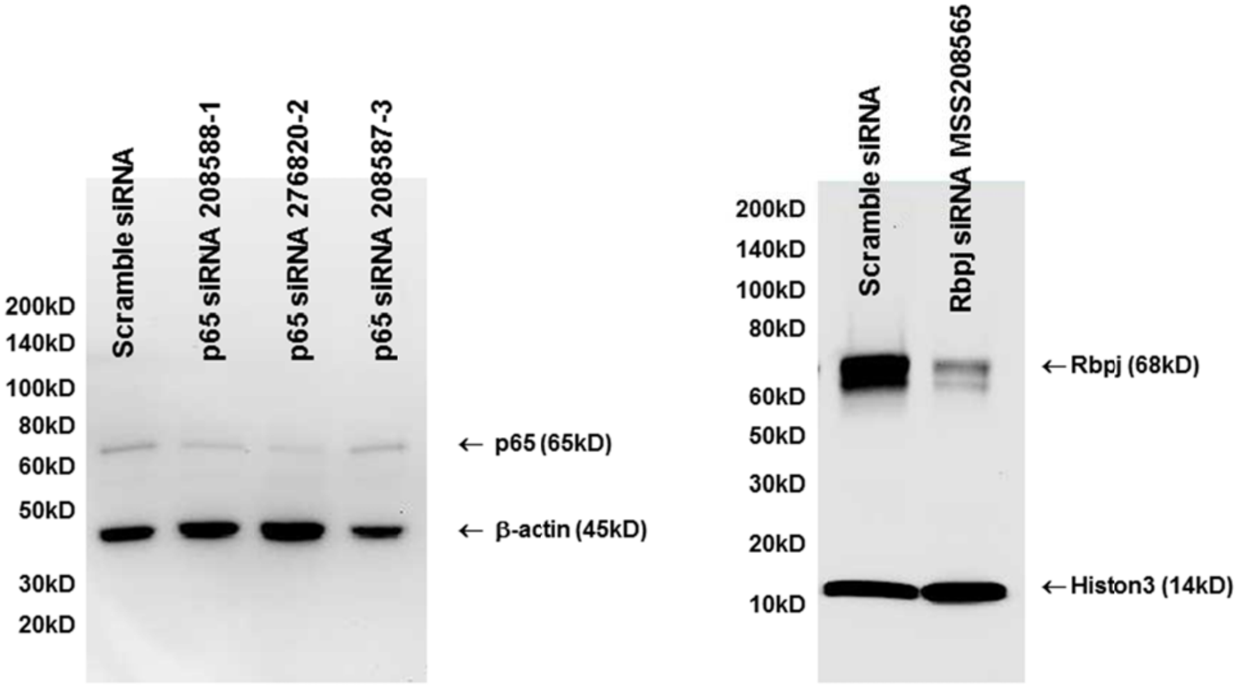

Supplementary Figure 16. Validation of siRNAs against p65 (left) and Rbpj (right) in mouse dermal endothelial cells by western blotting. For p65, siRNA276820 was used for experiments. For RBPJ, the nuclear fraction of cell lysates was used to validate the siRNA.

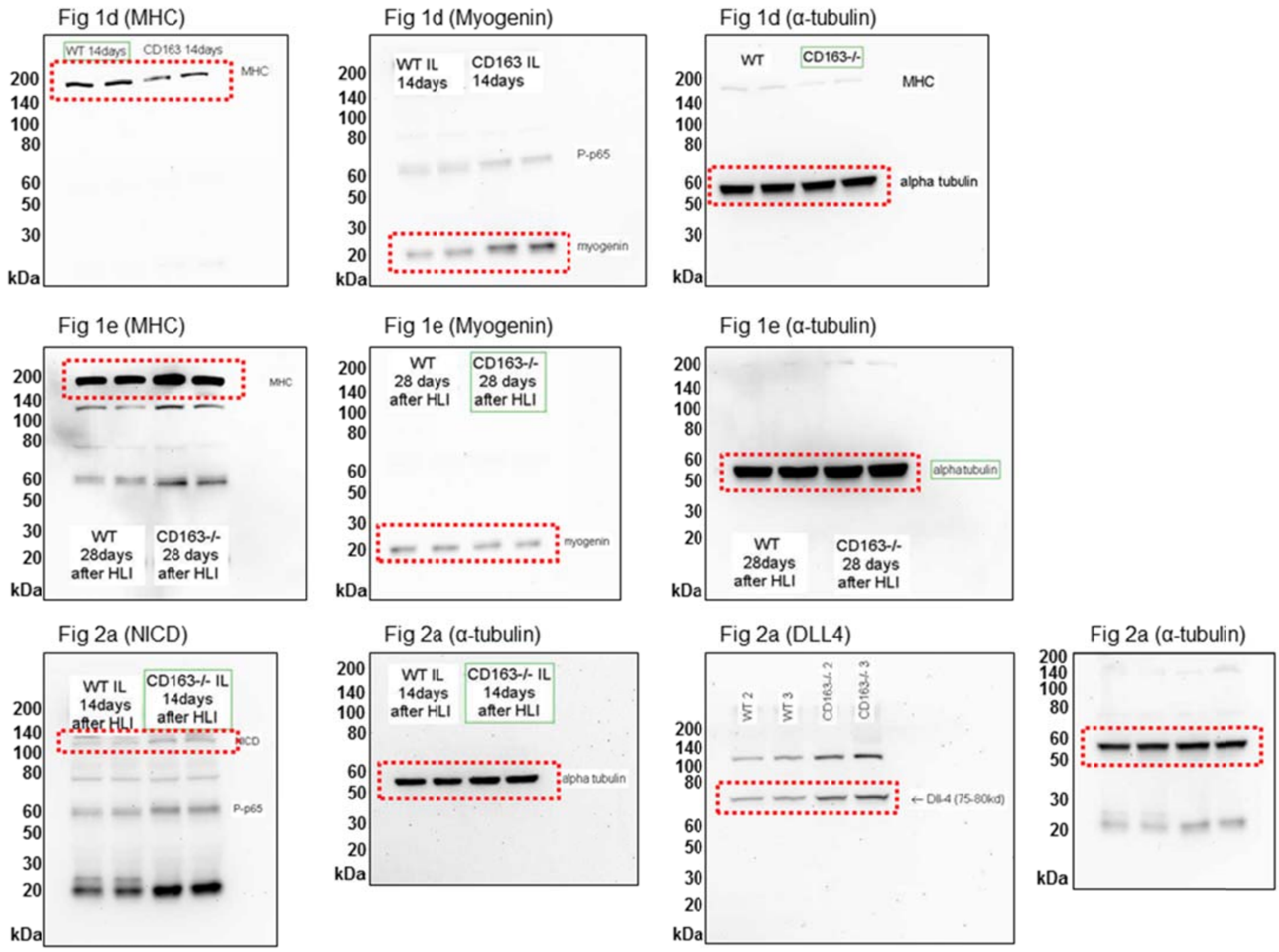

Supplementary Figure 17. Full immunoblots with indicated area of selection

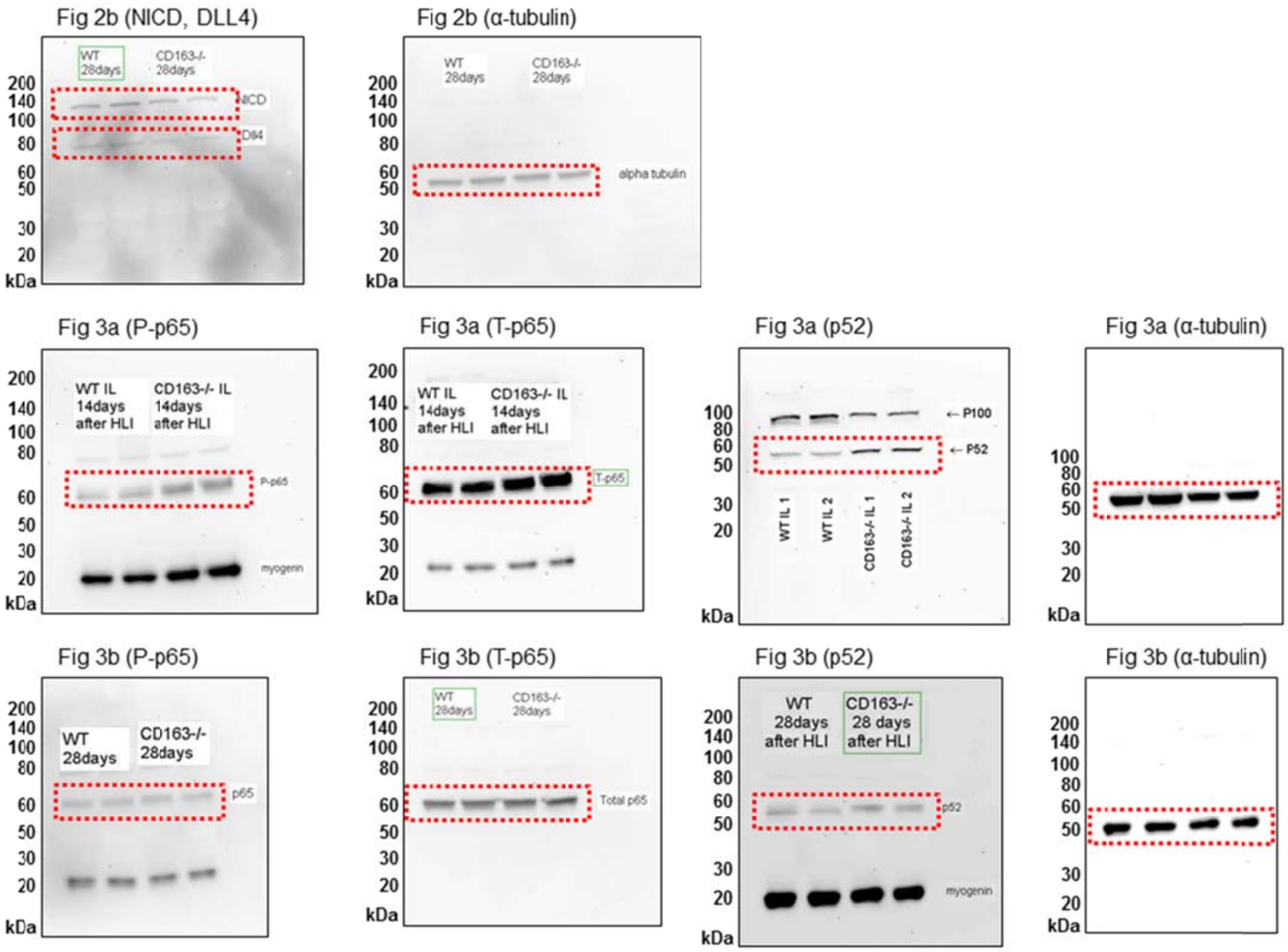

Supplementary Figure 18. Full immunoblots with indicated area of selection

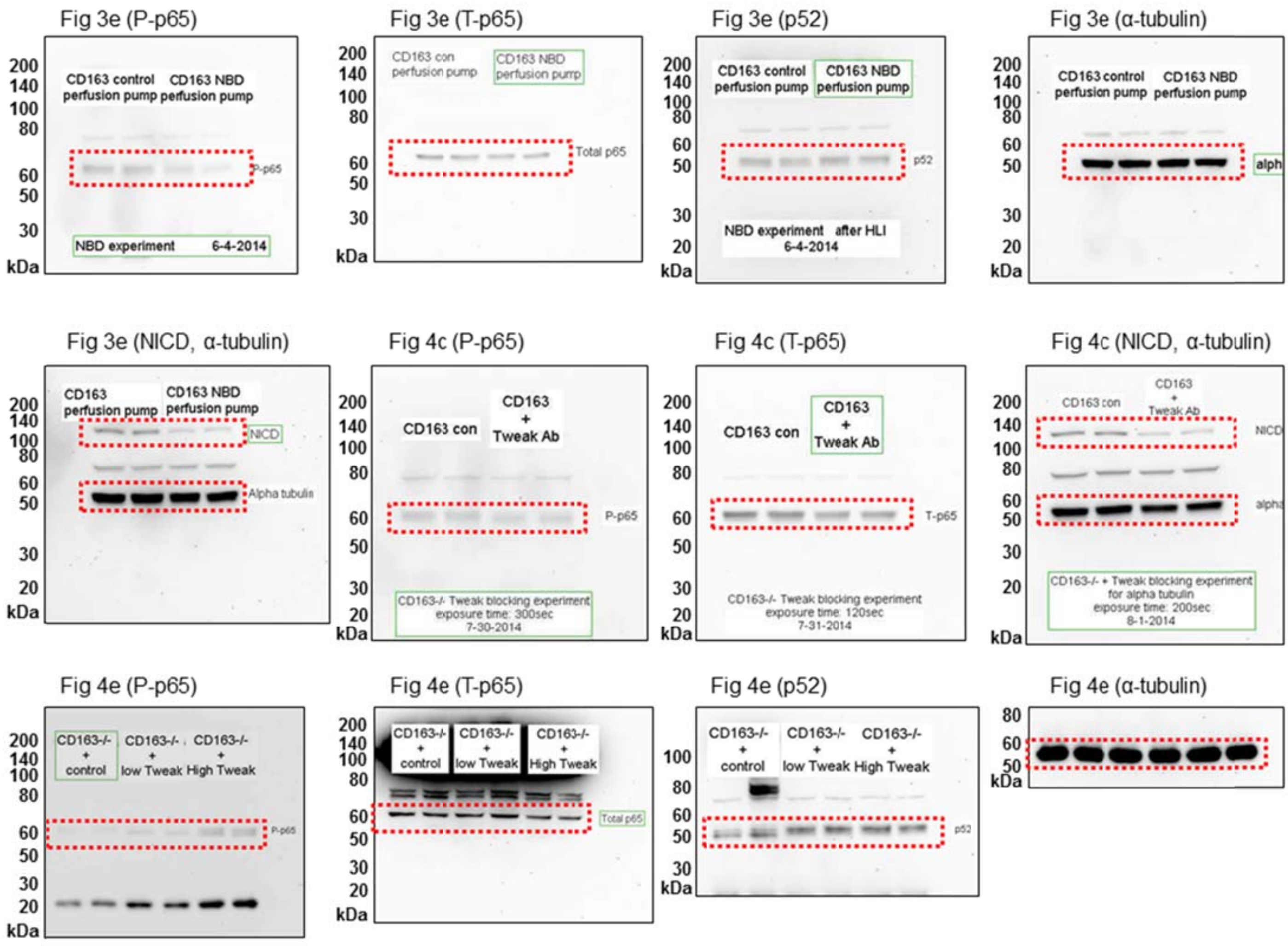

Supplementary Figure 19. Full immunoblots with indicated area of selection

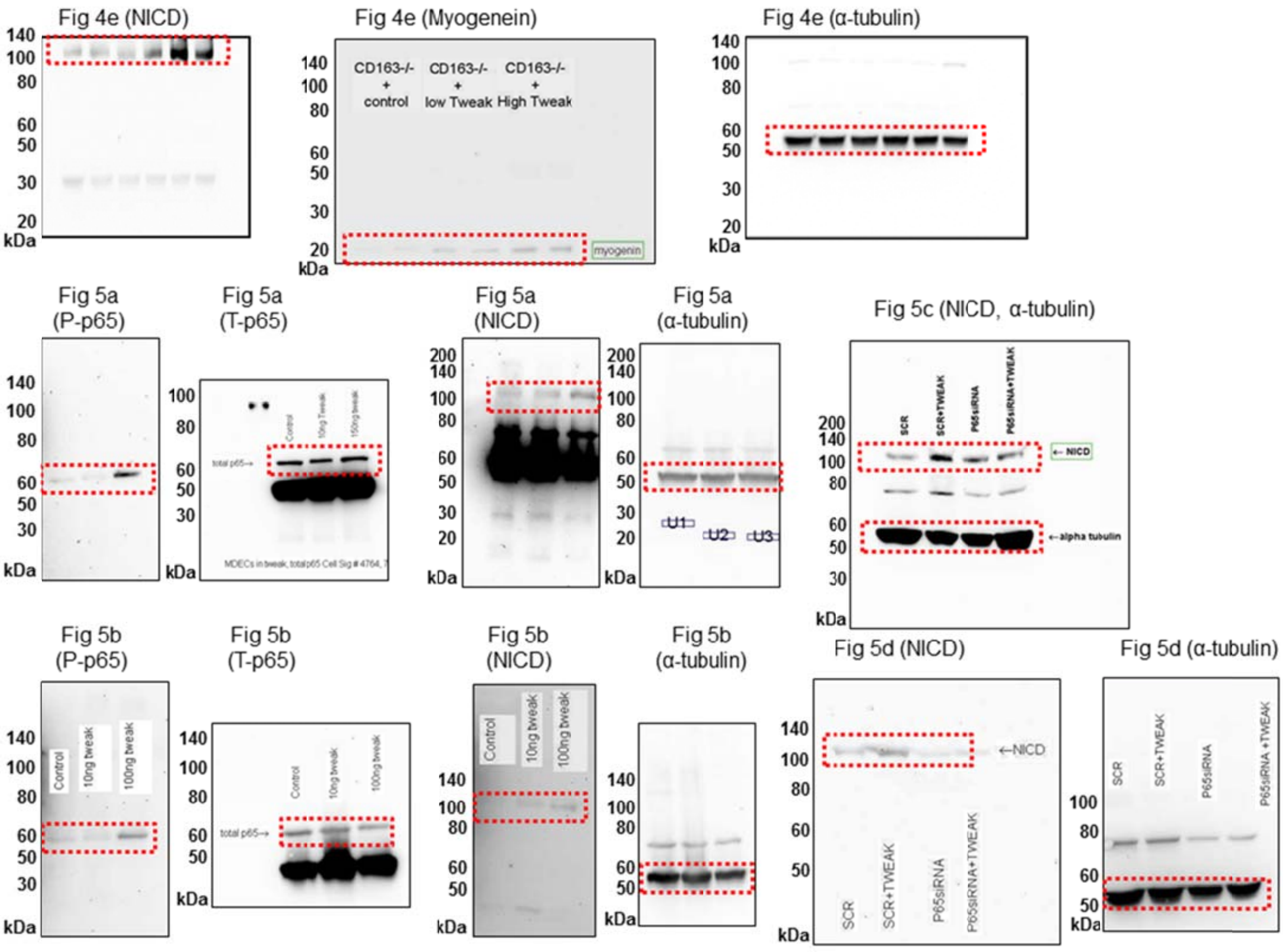

Supplementary Figure 20. Full immunoblots with indicated area of selection

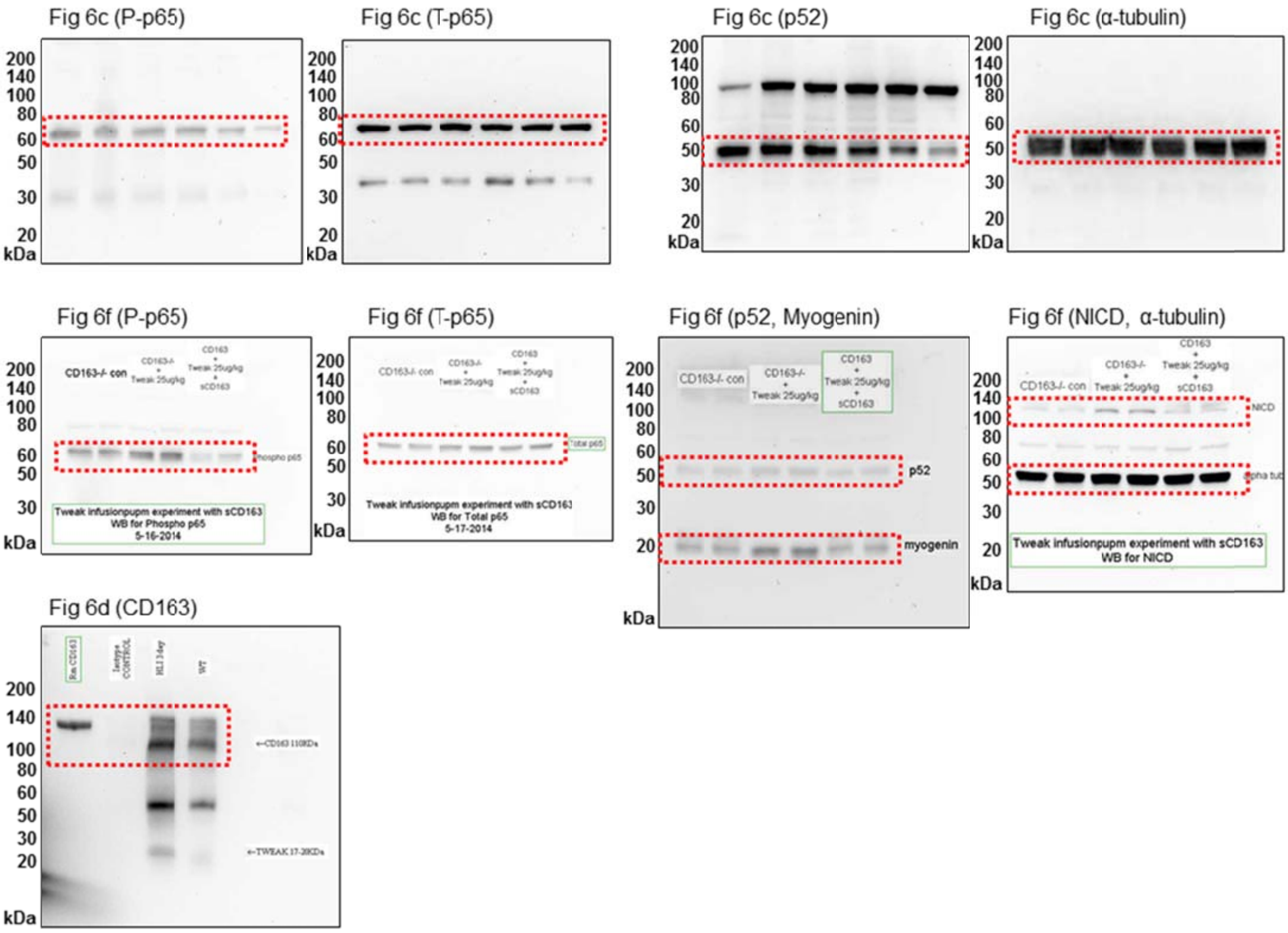

Supplementary Figure 21. Full immunoblots with indicated area of selection

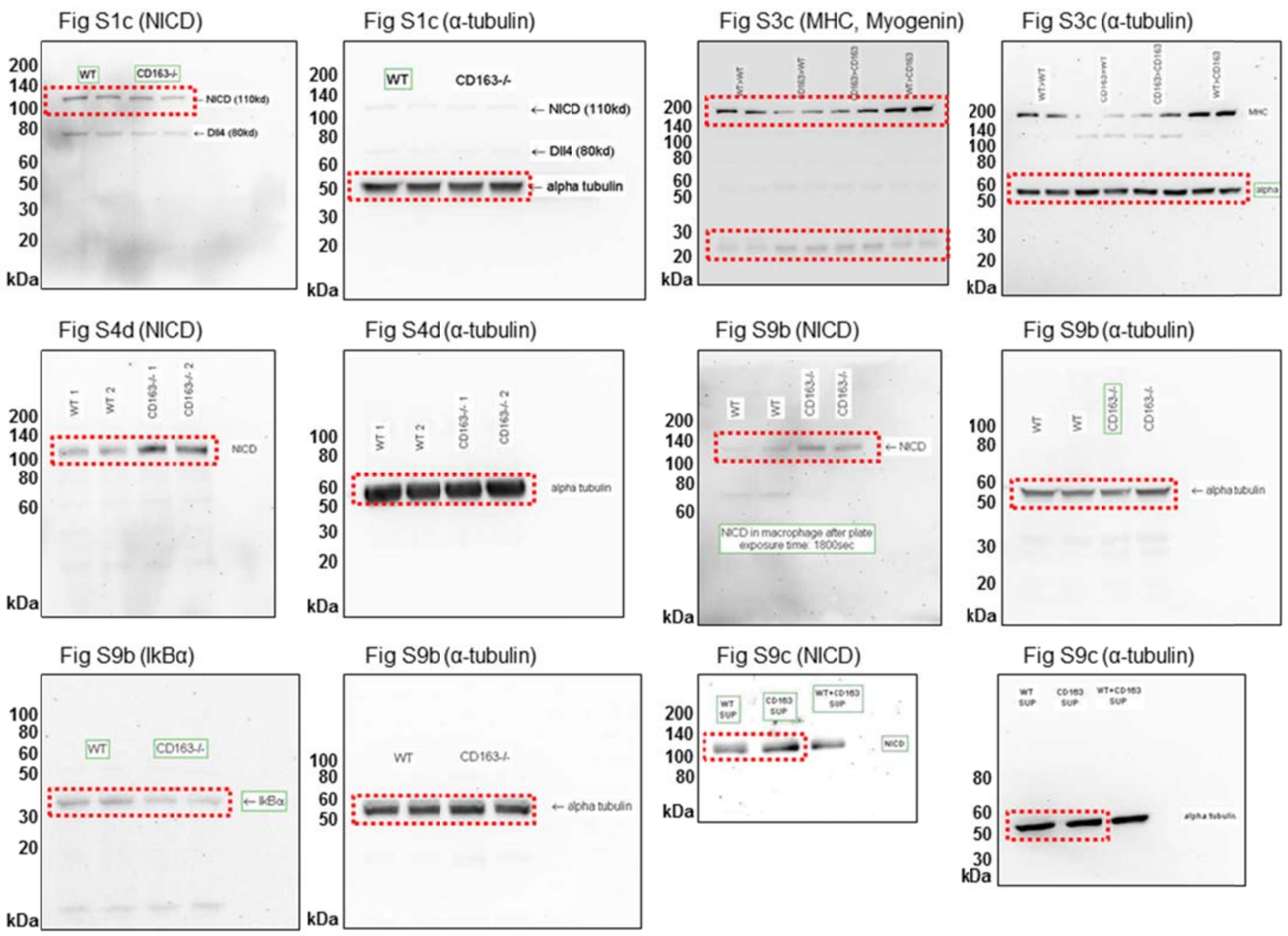

Supplementary Figure 22. Full immunoblots with indicated area of selection

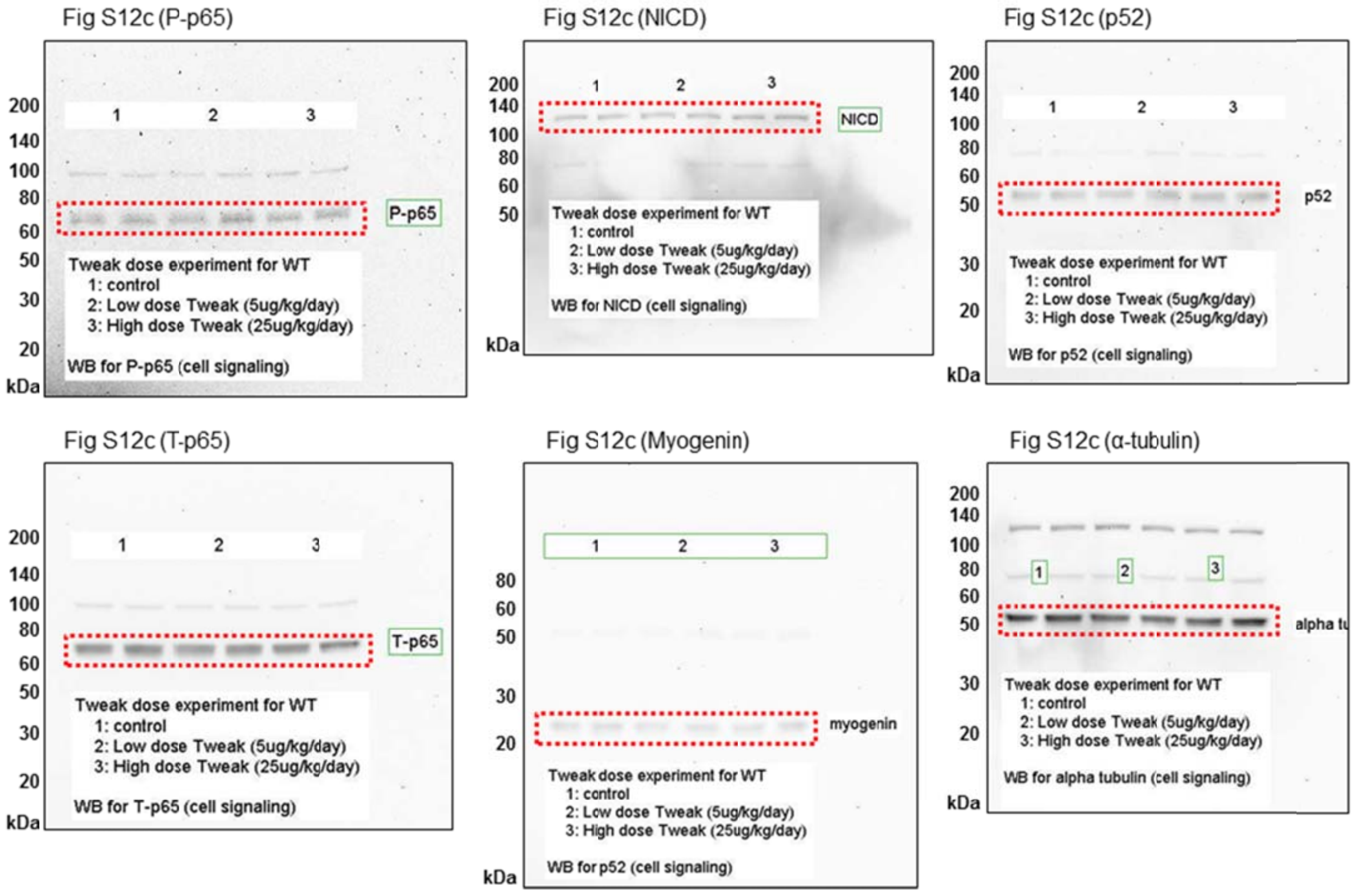

Supplementary Figure 23. Full immunoblots with indicated area of selection
